# Complementary Sensitivity of Fixed‐Time and Fixed‐Oscillation Regimes to Exchange and Structural Disorder in the Human Brain Revealed Using Oscillating‐Gradient Diffusion MRI With Ultra‐Strong Gradients

**DOI:** 10.1002/mrm.70300

**Published:** 2026-02-15

**Authors:** Dongsuk Sung, Kwok‐Shing Chan, Julianna Gerold, Wen Zhong, Jialan Zheng, Qiyuan Tian, Hua Guo, Susie Y. Huang, Hong‐Hsi Lee

**Affiliations:** ^1^ Athinoula A. Martinos Center for Biomedical Imaging, Department of Radiology Massachusetts General Hospital Charlestown Massachusetts USA; ^2^ Harvard Medical School Boston Massachusetts USA; ^3^ School of Biomedical Engineering, Tsinghua University Beijing China

## Abstract

**Purpose:**

Oscillating‐gradient spin‐echo (OGSE) diffusion MRI probes cell geometry and membrane integrity through the frequency‐dependence of kurtosis, but prior studies have reported inconsistent findings depending on how frequency is varied. We compared frequency‐dependent kurtosis in the human brain under two regimes: varying frequency with fixed total waveform duration (fixed‐T) or number of oscillations (fixed‐N).

**Methods:**

Eleven healthy volunteers were scanned on the 3 T Connectome 2.0 system with OGSE using 500 mT/m gradients. Mean kurtosis (MK) was measured using three fixed‐T waveforms (T ≈ 80 ms) and four fixed‐N waveforms (N = 1). The adiabatic Kärger exchange model was fit to MK in 48 white matter (WM) and 70 gray matter (GM) regions, yielding estimates of water exchange time ($t_{\mathrm{ex}}$), intracellular fraction ($f_{\mathrm{ic}}$), extracellular tortuosity ($\alpha_{D}$), and asymptotic kurtosis ($K_{\infty }$).

**Results:**

Distinct frequency‐dependence was observed between regimes. In fixed‐T, MK decreased with frequency in both WM and GM, reflecting diffusion coarse‐graining from structural disorder. In fixed‐N, MK increased with frequency in GM but was relatively flat in WM, indicating greater sensitivity to exchange. Parameter estimates showed biologically meaningful contrasts: WM exhibited longer $t_{\mathrm{ex}}$, higher $f_{\mathrm{ic}}$, and greater $\alpha_{D}$, consistent with tightly packed, myelinated axons and anisotropic extracellular space. GM showed shorter $t_{\mathrm{ex}}$ and higher $K_{\infty }$, reflecting greater heterogeneity and CSF partial volume.

**Conclusion:**

Fixed‐T and fixed‐N OGSE provide complementary sensitivity to microstructural features. Fixed‐T emphasizes structural disorder, while fixed‐N highlights membrane permeability and exchange. The extended frequency regimes enabled by ultra‐strong gradients advance OGSE as a powerful tool for probing human brain tissue microstructure.

## 1 Introduction

Diffusion MRI (dMRI) measures the random motion of water molecules in brain tissue, shaped by obstacles such as cell membranes, dendrites, cell bodies, and glial processes [1] and provides biologically meaningful markers of cellular organization and membrane integrity with applications in stroke [2, 3], Alzheimer's disease [4], and brain tumors [5, 6]. In biological tissue, water diffusion rarely follows a purely Gaussian pattern, since microstructural barriers introduce restrictions and heterogeneity. These non‐Gaussian diffusion processes, as captured by nonzero kurtosis and by the dependence of diffusivity and kurtosis on diffusion time or frequency [7, 8], carry information about features such as cell size and transmembrane exchange that simple Gaussian models cannot capture. Importantly, both diffusivity and kurtosis depend on the temporal scale of diffusion encoding, which can be characterized by applying diffusion tensor imaging (DTI) [9] and diffusion kurtosis imaging (DKI) [10, 11], respectively. Diffusivity generally shows a monotonic trend, decreasing with longer diffusion times or increasing with higher frequencies, consistent with diffusion coarse‐graining [8]. In contrast, kurtosis does not always follow a simple monotonic pattern. Instead, theory predicts [12] and experiments confirm [13, 14, 15, 16, 17] that kurtosis can vary non‐monotonically with diffusion time and frequency in neuronal tissues.

**FIGURE 1:**
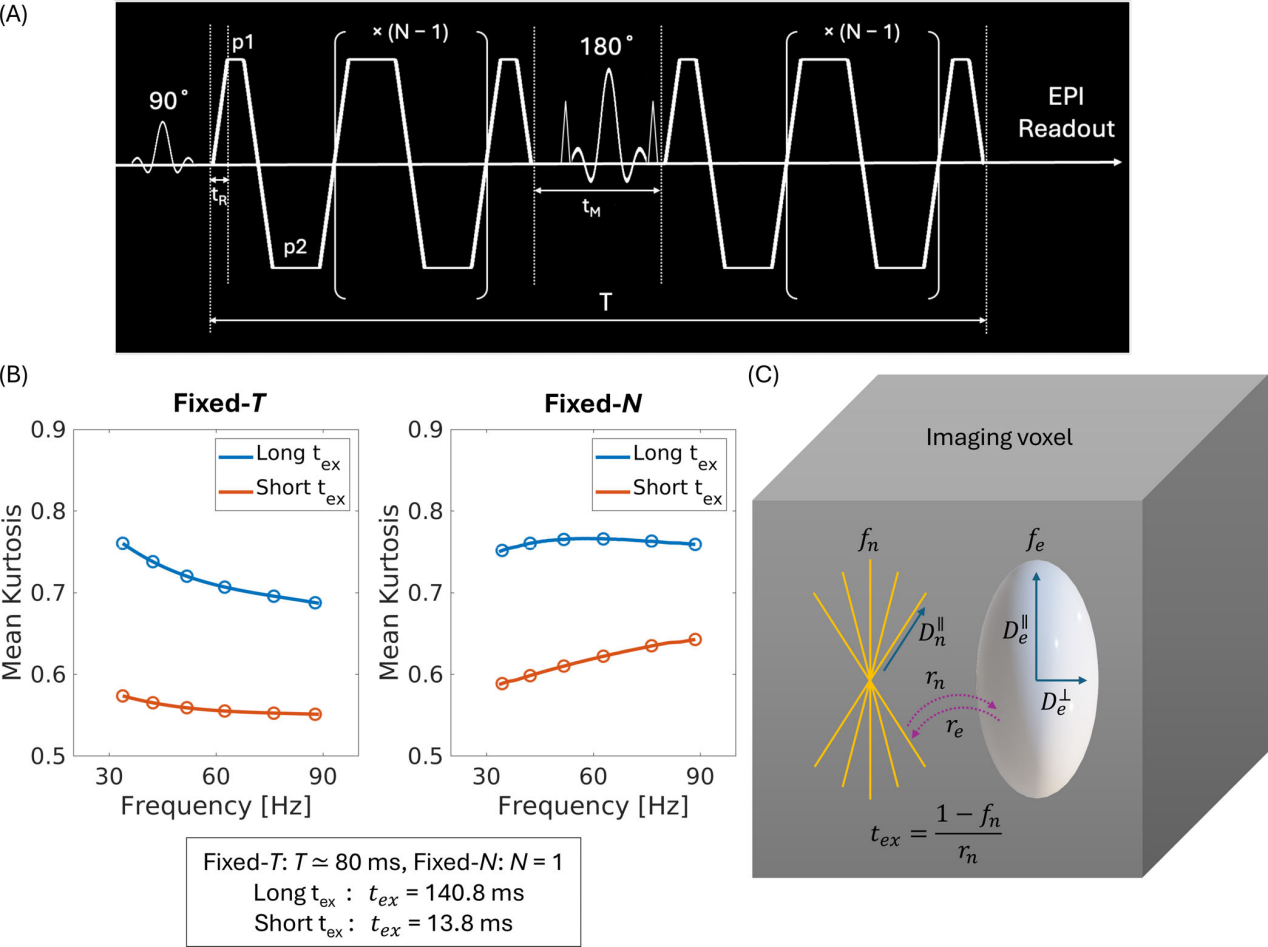
(A) Oscillating‐gradient waveform used in this study, consisting of two symmetric groups of trapezoidal cosine lobes with N oscillations on either side of the refocusing RF pulse. Total waveform duration T is determined by the sum of the plateau times (p1, p2), rise time ($t_{R}$), and mixing time ($t_{M}$). (B) Prior simulations and preclinical studies revealed distinct frequency‐dependence of kurtosis in the fixed‐T and fixed‐N regimes [12, 24]. The blue curves represent tissue with long exchange time $t_{\mathrm{ex}}$ = 140.8 ms, and orange curves represent tissue with relatively short $t_{\mathrm{ex}}$ = 13.8 ms. Mean kurtoses were simulated at sampled frequencies of 34.0, 42.2, 51.6, 67.2, 76.6, and 88.3 Hz (blue and orange circles), and the rest of the data points were nonlinearly interpolated (spline). (C) Schematic of the two‐compartment microstructural model used in this study, comprising intra‐neurite and extracellular spaces. Key parameters include intra‐neurite diffusivity ($D_{n}^{∥}$), extracellular diffusivities parallel and transverse to neurites ($D_{e}^{∥}$, $D_{e}^{⊥}$), intra‐neurite signal fraction ($f_{n}$), and inter‐compartmental water exchange time ($t_{\mathrm{ex}}$).

Oscillating‐gradient spin‐echo (OGSE) sequences, which apply multiple cycles of diffusion gradient pairs, has been introduced to probe diffusion at shorter effective time and length scales by using higher oscillation frequencies [6, 18, 19, 20, 21, 22]. Recent work has demonstrated frequency‐dependent kurtosis at effective diffusion times as short as ˜10 ms in the human brain [15, 23]. The effective diffusion time probed by OGSE is inversely proportional to oscillation frequency, which can be tuned by two parameters: the number of oscillation cycles (N) and total waveform time (T) (Figure 1A). Given that the frequency of oscillating‐gradient waveform can be approximately expressed as ω/2π˜N/T, the frequency can be varied by either (i) changing N with a fixed T (fixed‐T regime) or (ii) changing T with a fixed N (fixed‐N regime).

Most prior in vivo OGSE studies have used the fixed‐T regime, which facilitates stronger diffusion‐weighting [15, 23, 25, 26, 27, 28, 29], while keeping the echo times consistent across frequencies. For example, Dai et al. explored kurtosis frequency dependence in the healthy living human brain [23] and observed a monotonic decrease in kurtosis with increasing oscillating frequency from 0 Hz (representing pulsed‐gradient waveform) to 47.5 Hz in both gray and white matter (GM and WM). In contrast, simulations and animal studies using the fixed‐N regime have shown a non‐monotonic or biphasic alteration of kurtosis frequency dependence [14, 24]. These earlier studies were largely descriptive and did not apply biophysical models for tissue‐specific interpretation. In our own prior simulations, we demonstrated systematic differences between regimes: monotonic kurtosis decrease with frequency under fixed T, and non‐monotonic behavior under fixed N (Figure 1B) [12]. These patterns can be understood using a two‐compartment exchange model that accounts for restricted intracellular diffusion [30, 31, 32].

At the tissue level, the human brain can be viewed as a mixture of neurites (axons and dendrites), somas (cell bodies), and extracellular space (Figure 1C). Kurtosis dynamics with time or frequency may reflect both structural disorder within compartments (e.g., beadings along neurites) and inter‐compartmental exchange between intra‐ and extracellular water [12, 15, 24, 33]. Prior models have considered these two sources separately. For example, the two‐pool exchange model can be formulated using coupled Bloch–Torrey equations, with isotropic or anisotropic Gaussian diffusion in each compartment and water exchange between them [30, 34]. This so‐called Kärger model is applicable when the diffusion time scale is sufficiently long that diffusion in each compartment is approximately Gaussian, with negligible diffusivity time‐dependence. At intermediate time scales, however, the diffusivity of each compartment shows nontrivial time‐ or frequency‐dependence due to microstructural hindrance [8, 33, 35] and should be accounted for when modeling the kurtosis dynamics. More recent extensions incorporate effective medium theory (EMT) into the Kärger model in the frequency domain [36]. Nevertheless, the functional form of kurtosis is complicated and challenging to adapt to experimental oscillating‐gradient waveforms, which typically have trapezoidal shapes, finite cycles, and nonzero mixing times to accommodate the refocusing RF pulse between the two diffusion‐encoding blocks (Figure 1A). To address this, we used an adiabatic extension of the generalized Kärger model that accounts for both compartment‐specific frequency‐dependence and realistic gradient waveforms [12, 37].

In this study, we acquired in vivo OGSE dMRI in 11 healthy volunteers on the Connectome 2.0 scanner with gradient strengths up to 500 mT/m and relatively conservative slew rates up to 300 T/m/s for subject comfort [38, 39]. This study represents one of the earliest in vivo human applications of the adiabatic Kärger model to diffusion data acquired with ultra‐high gradients. To our knowledge, the investigation of kurtosis frequency‐dependence across distinct frequency‐varying regimes, and its potential for disentangling tissue parameters, remains limited. Furthermore, compared with the previous study of adiabatic Kärger model [12], we additionally incorporated the diffusivity frequency‐dependence of hindered diffusion due to structural disorders [8] in the extracellular space, with consideration of trapezoidal cosine gradient waveform and optimized mixing time ($t_{M}$) in experiments, cf. ideal cosine waveform. We hypothesized that inter‐compartmental water exchange underlies the distinct frequency dependence of kurtosis between the two regimes, and that the orthogonal patterns they reveal can provide complementary sensitivity to microstructural parameters such as exchange time, intra‐neurite signal fraction, and extracellular tortuosity.

## 2 Theory

The frequency‐dependence of kurtosis arises primarily from two microstructural mechanisms: (1) structural disorder within neurites, such as caliber variations or beadings along neurites, described by EMT [8] and (2) water exchange between intra‐ and extracellular spaces, modeled by the anisotropic adiabatic Kärger model [30]. When both mechanisms are considered, for a linear‐tensor‐encoding gradient waveform, the frequency‐dependent mean kurtosis (MK) is expressed in a generalized Kärger model [37]:

(1) $$\bar{K}={\bar{K}}_{\mathrm{var}}\cdot h\left[q\right]+K_{\infty },$$

where

$$K_{\mathrm{var}}\equiv \frac{3\mathrm{var}\left(D\right)}{{\left\langle D\right\rangle }^{2}},$$

(2) $${\bar{K}}_{\mathrm{var}}=\int_{0}^{1}K_{\mathrm{var}}\left(\xi \right)\;d\xi ,$$

with ⟨D⟩ and var(D) denote the mean and variance of diffusivities across compartments, h[q] is a functional related to water exchange between two compartments [37], and $K_{\infty }$ is the asymptotic kurtosis value attributed to the variance of long‐time diffusivities from other non‐exchanging compartments (e.g., CSF) due to the partial volume effect [33]. $K_{\mathrm{var}}$ corresponds to the direction‐dependent apparent kurtosis in the absence of exchange and partial volume effect, and ${\bar{K}}_{\mathrm{var}}$ is directionally averaged over $\xi =\hat{g}\cdot \hat{n}$ with the diffusion gradient direction $\hat{g}$ and neurite direction $\hat{n}$.

### 2.1 Structural Disorder

The classical Kärger model assumes Gaussian diffusion within two exchanging compartments and therefore does not capture frequency‐dependent diffusivity [30]. To account for the diffusivity frequency‐dependence from structural disorder through $K_{\mathrm{var}}$, we use an adiabatic extension of the Kärger model [12], where the diffusivity in each compartment depends on frequency sufficiently slowly, such that we can use their frequency‐dependent values to calculate $K_{\mathrm{var}}$ (Equation 2) as if they are constants [30]. In this framework, the compartmental diffusivities and their variance are:

$$\left\langle D\right\rangle =f_{\mathrm{ic}}D_{\mathrm{ic}}+\left(1-f_{\mathrm{ic}}\right)D_{\mathrm{ec}},$$

(3) $$\mathrm{var}\left(D\right)=f_{\mathrm{ic}}\left(1-f_{\mathrm{ic}}\right){\left(D_{\mathrm{ec}}-D_{\mathrm{ic}}\right)}^{2}.$$

where $f_{\mathrm{ic}}$ is the intracellular volume fraction, and $D_{\mathrm{ic}}$ and $D_{\mathrm{ec}}$ are the (direction‐dependent) intra‐ and extracellular diffusivities. Considering the anisotropy in each compartment, we decompose $D_{\mathrm{ic}}$ and $D_{\mathrm{ec}}$ into components parallel and transverse to the neurites with the assumption that intra‐ and extracellular spaces are aligned to one another. Based on the thin diameter of neurites (∼ 1 μm) [40], we assumed the intracellular transverse diffusivity is small and negligible ($D_{\mathrm{ic}}^{⊥}/D_{\mathrm{ic}}^{∥}\ll 1$). Furthermore, the time‐dependence of transverse diffusivity in the extracellular space is small and challenging to observe in the human brain [41]; thus, we expect that its frequency dependence is also small and approximates the extracellular transverse diffusivity $D_{\mathrm{ec}}^{⊥}$ as its long‐time bulk value $D_{\infty }^{⊥}$, that is, $D_{\mathrm{ec}}^{⊥}≃D_{\infty }^{⊥}$. Finally, to avoid the degeneracy problem in model fitting, we assume that the parallel diffusivities of the intra‐ and extracellular spaces are the same, that is, $D_{\mathrm{ic}}^{∥}=D_{\mathrm{ec}}^{∥}$. Under these assumptions, we express the direction‐dependent apparent diffusivity of the intra‐ and extracellular spaces as

$$D_{\mathrm{ic}}=D_{\mathrm{ic}}^{∥}\xi^{2},$$

(4) $$D_{\mathrm{ec}}=D_{\mathrm{ic}}^{∥}\xi^{2}+D_{\infty }^{⊥}\left(1-\xi^{2}\right).$$

Based on EMT, structural disorder, such as caliber variations (or beadings) along neurites, leads to an intracellular parallel diffusivity $D_{\mathrm{ic}}^{∥}$ approaching to its long‐time bulk value $D_{\infty }^{∥}$ with the frequency (ω)‐dependence $∼\sqrt{\omega }$ for an OG sequence of ideal cosine‐waveform [8, 25, 33, 35]. To account for experimental OG waveforms (trapezoidal shape, finite oscillation number N and waveform time T, Figure 1A), we apply the Gaussian phase approximation (Supporting Information Theory), yielding the intracellular parallel diffusivity

(5) $$D_{\mathrm{ic}}^{∥}=D_{\infty }^{∥}+cV_{\sqrt{\omega }},$$

where c is the strength of restrictions related to the mean and variance of the distance between local swellings/bead‐like structures along neurite (the disorder in the bead positions) and the dimensionless bead fraction (the prominence of the beads) [42], $V_{\sqrt{\omega }}=\frac{1}{b}\int \frac{d\omega }{2\pi }{\left|q_{\omega }\right|}^{2}\sqrt{\left|\omega \right|}$, $b=\int \frac{d\omega }{2\pi }{\left|q_{\omega }\right|}^{2}$ is the diffusion weighting, and $q_{\omega }$ is the Fourier transform of $q\left(t\right)=\int_{0}^{t}\gamma G\left(t^{\prime }\right)dt^{\prime }$ with diffusion gradient waveform G(t) and gyromagnetic ratio γ. Substituting Equations ((3), (4), (5)) into apparent kurtosis (Equation 2) and averaging over all directions, we obtain the MK with the absence of water exchange:

(6) $${\bar{K}}_{\mathrm{var}}=\int_{0}^{1}d\xi \;3f_{\mathrm{ic}}\left(1-f_{\mathrm{ic}}\right)\cdot {\left[\alpha_{D}+\alpha_{C}V_{\sqrt{\omega }}\frac{\xi^{2}}{1-\xi^{2}}+1-f_{\mathrm{ic}}\;\right]}^{-2},$$

where $\alpha_{D}=D_{\infty }^{∥}/D_{\infty }^{⊥}$ is the extracellular tortuosity, and $\alpha_{C}=c/D_{\infty }^{⊥}$ is the frequency‐dependent factor.

### 2.2 Water Exchange

The contribution of water exchange to overall kurtosis frequency‐dependence (Equation 1) is modulated by an isotropic exchange sensitivity functional h[q] in Kärger model, given by [37]

(7) $$h\left[q\right]=2\int_{0}^{T}e^{-t/t_{\mathrm{ex}}}q_{4}\left(t\right)dt,$$

where T is the total waveform time, $q_{4}\left(t\right)=\frac{1}{b^{2}}\int_{0}^{T}q^{2}\left(t^{\prime }\right)q^{2}\left(t^{\prime }+t\right)\;dt^{\prime }$, $b=\int_{0}^{T}q^{2}\left(t\right)\;dt$ is the diffusion weighting, and $t_{\mathrm{ex}}$ is the water exchange time between the intra‐ and extracellular spaces.

### 2.3 Model Parameter Estimation in Frequency‐Dependent Mean Kurtosis

Finally, to link model predictions to experimental data, we fit the adiabatic Kärger model in Equations (1), (6), and (7) to the measured MK (${\bar{K}}_{\text{meas}}$) for each gradient waveform G(t) with known sequence parameters (T, N, $t_{M}$) and estimate five tissue parameters, including $t_{\mathrm{ex}}$, $f_{\mathrm{ic}}$, $\alpha_{D}$, $\alpha_{C}$, and $K_{\infty }$. The model parameters were estimated by solving the following optimization problem with L_1_‐norm:

(8) $${\text{argmin}}_{t_{\mathrm{ex}},f_{\mathrm{ic}},\alpha_{D},\alpha_{C},K_{\infty }}{\left\|\bar{K}\left(T,N,t_{M};t_{\mathrm{ex}},f_{\mathrm{ic}},\alpha_{D},\alpha_{C},K_{\infty }\right)-{\bar{K}}_{\text{meas}}\right\|}_{L_{1}}.$$

## 3 Methods

### 3.1 In Vivo MR Acquisition

**FIGURE 2:**
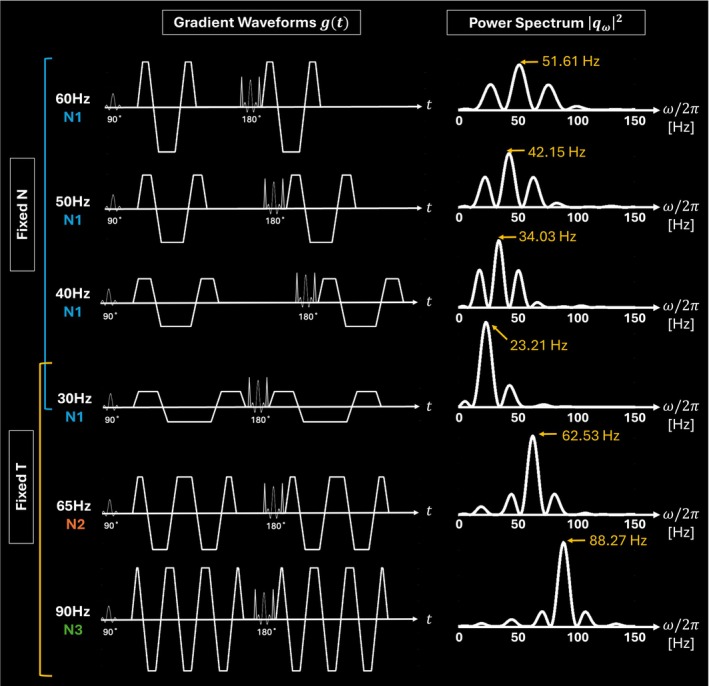
Gradient waveform g(t) and corresponding power spectrum ${\left|q_{\omega }\right|}^{2}$ for each oscillating‐gradient waveform in fixed‐N (the number of oscillations) and fixed‐T (total waveform time) regimes. The peak frequency for each power spectrum is indicated with a yellow arrow pointing to its location.

This study was approved by the local institutional review board. All healthy volunteers provided written informed consent prior to participation. Eleven participants (mean age = 27 ± 5 years, 6 females) underwent multi‐shell dMRI using OGSE (Figure 1A) on the 3 T Connectome 2.0 MRI scanner (MAGNETOM Connectom.X, Siemens Healthineers, Erlangen, Germany) equipped with a maximum gradient strength of 500 mT/m and slew rate of 300 T/m/s [38, 39]. A custom‐built 72‐channel in vivo head coil was used for signal reception [43]. Two frequency‐varying regimes were implemented by (i) varying the number of oscillations (fixed‐T regime) and (ii) varying total waveform time (fixed‐N regime) (Figure 2, Table 1). N is defined as the number of trapezoidal diffusion‐encoding gradient lobes on each side of the 180° refocusing pulse, with the first ¾ cycle and last ¼ cycle encoded separately and the middle (N−1) cycles repeated. T is defined as the total time from the beginning of the first encoding block (before the refocusing pulse) until the end of the second encoding block (after the refocusing pulse).

**TABLE 1:** Input and derived parameters of oscillating‐gradient waveforms. Total waveform time, peak frequency of the power spectrum, and mixing time for each acquisition. Mixing times were optimized by maximizing the peak amplitude of the power spectrum at the true frequency.

Fixed regime	*T*	*T*	*T* and *N*	*N*	*N*	*N*
Input frequency, *ω*_*i*_/2π (Hz)	90	65	30	40	50	60
Number of oscillations, *N*	3	2	1	1	1	1
Mixing time, *t*_*M*_ (ms)	10.1	15.2	7.4	30.9	24.7	20.4
Total waveform time, *T* (ms)	80.0	79.9	77.3	84.1	67.9	56.9
Peak frequency, *ω*_*p*_/2π (Hz)	88.3	62.5	23.2	34.0	42.2	51.6

Mixing times were optimized by maximizing the ratio of “peak amplitude” and “side lobe amplitude” of each corresponding power spectrum for each gradient waveform (see Supporting Information Methods 1 for more details), which is similar to previous literature maximizing the peak of the gradient waveform power spectrum at the target frequency [26]. In the case of 30 Hz with *N* = 1 (30 Hz‐N1, we will refer to each waveform by this format throughout the manuscript); however, the optimal $t_{M}$ was too long for a reasonable echo time (TE), thus the minimum scanner‐allowed $t_{M}$ (7.4 ms) was used. Other parameters included *G*_max_ = 500 mT/m, *SR*_max_ = 300 T/m/s (relatively conservative choice to avoid subject's discomfort in the scanner and the potential risk of peripheral nerve stimulation), two *b*‐shells= [1, 2] ms/μm^2^ with 20 diffusion directions per shell, TE/TR = 118/7600 ms, 2 mm isotropic voxel size, GRAPPA = 2, PF = 6/8, SMS = 2. To achieve the maximum *b*‐value of 2 ms/μm^2^, gradient amplitudes of 137, 213, 303, 405, 326, and 465 mT/m were used for gradient waveforms of 30 Hz‐N1, 40 Hz‐N1, 50 Hz‐N1, 60 Hz‐N1, 65 Hz‐N2, and 90 Hz‐N3, respectively. The same TE (118 ms) was used for all gradient waveforms. See Supporting Information Methods 2 for more details about frequency selection and the impact of optimized mixing time.

### 3.2 Diffusion MRI on Water Phantom

To investigate the potential influence of eddy currents or mechanical vibrations on MK measurement, we conducted a scan in a pure water bottle phantom using the same OGSE waveforms (30 Hz‐N1, 40 Hz‐N1, 50 Hz‐N1, 60 Hz‐N1, 65 Hz‐N2, and 90 Hz‐N3) as the in vivo acquisition protocol (Section 3.1) for multi‐shell dMRI on the 3 T Connectome 2.0 MRI scanner (MAGNETOM Connectom.X, Siemens Healthineers, Erlangen, Germany) [38, 39] with the 72‐channel in vivo head coil [43]. A distilled water phantom (inner bottle) was surrounded with ice (outer bottle) (Figure S1A) to minimize the effect of temperature changes during the scan. We set a 30 × 30 × 30 mm^3^ cube region of interest (ROI) at the center of the inner water bottle (a blue rectangle over *b* = 0 image in axial view, Figure S1B).

### 3.3 Image Processing and Diffusion and Kurtosis Tensor Estimation

A modified DESIGNER pipeline was used to preprocess both in vivo and phantom dMRI data [44]. The pipeline began with denoising using Rician‐noise‐floor‐corrected Marchenko–Pastur principal component analysis (MP‐PCA) with 10 iterations [45, 46], followed by Gibbs ringing artifact removal from MRtrix3 [47, 48], TOPUP from FSL (v6.0.7) [49], EDDY from FSL (v6.0.7) [50], and gradient nonlinearity correction using in‐house code [51, 52]. The preprocessed data went through diffusion and kurtosis tensor estimation in MRtrix3 (v3.0.3) [48] using weighted linear least squares and iterative weighted linear least squares, and the fitting was constrained to nonnegative diffusivity and kurtosis as well as monotonic signal decay [53, 54, 55]. Given that physiologically reasonable MK was within the range of 0 ≤ MK ≤ 3 [56, 57], voxels with MK < 0 and MK > 3 were excluded from the analysis.

### 3.4 ROIs Analysis

Microstructure parameters were estimated within small‐to‐large scale ROIs in GM and WM. Using a mean *b* = 0 map from dMRI, 32 subcortical and 68 cortical ROIs were acquired by Synthseg [58] based on FreeSurfer subcortical segmentation labels [59] and Desikan–Killiany atlas‐derived cortical parcellation labels [60, 61]. All segmented and parceled labels were extracted and co‐registered to each subject's dMRI space using MRtrix3 (v3.0.3) [51]. Among the GM ROIs, 14 subcortical ROIs and 56 cortical ROIs were selected or combined as a larger ROI (e.g., combined pars opercularis, pars triangularis, and pars orbitalis into inferior frontal). Global cortex was acquired by combining all 56 cortical ROIs into a single large ROI. Global WM (or cerebral WM) was defined based on FreeSurfer subcortical segmentation labels [59]. However, for smaller WM ROIs, a JHU ICBM label map with 48 labels from JHU DTI‐based WM atlases [62] was registered to each subject's dMRI space based on symmetric normalization with rigid + affine + deformable transformation using ANTs [63]. For each ROI, mean diffusivity and MK values were extracted, and the median across voxels within the ROI was used for microstructural parameter fitting.

### 3.5 Microstructure Parameter Estimation

Based on the adiabatic Kärger model in Equations (1), (6), and (7), five microstructure parameters were estimated using the GPU‐accelerated, minimization‐based optimization framework (askAdam) in the GACELLE toolbox [64, 65, 66] within each ROI for each subject. GPU‐based acceleration was used to run askAdam in all ROIs simultaneously. The L1 loss function in Equation (8) was used to solve the optimization problem. A forward model for microstructure parameter fitting based on Equations (1), (6), and (7) generated mean kurtoses for all six gradient waveforms and across all ROIs during each optimization cycle using element‐wise arithmetic operations to derive the loss in Equation (8). Starting points for the optimization were set differently in WM and GM ROIs. In WM ROIs, parameters were initialized at $t_{\mathrm{ex}}$ = 150 ms, $f_{\mathrm{ic}}$ = 0.7, $\alpha_{D}$ = 2.5, $\alpha_{C}$ = 5 ms^1/2^, and $K_{\infty }$ = 0.1. In GM ROIs, parameters were initialized at $t_{\mathrm{ex}}$ = 20 ms, $f_{\mathrm{ic}}$ = 0.4, $\alpha_{D}$ = 2, $\alpha_{C}$ = 10 ms^1/2^, and $K_{\infty }$ = 0.4. The starting points were decided based on reported values of the model parameters from previous studies [33, 67, 68]. Fitting boundary of each microstructure parameter was the same for WM and GM ROIs: $t_{\mathrm{ex}}\in$ [1, 200] ms, $f_{\mathrm{ic}}\in$ [0.01, 0.99], $\alpha_{D}\in$ [1, 10], $\alpha_{C}\in$ [0.01, 20] ms^1/2^, and $K_{\infty }\in$ [0.01, 1].

### 3.6 Assessment of Mean Kurtosis Prediction Using Fitted Model Parameters

To evaluate the quality‐of‐prediction, we compared measured MK values with those predicted by the model and fitted parameters based on the Pearson's correlation coefficient (R), root mean square error (RMSE), and Lin's concordance correlation coefficient (CCC) [69] with a density scatter plot and a Bland–Altman plot for all 11 subjects, 6 gradient waveforms, and 118 ROIs (both left and right hemisphere ROIs). Lin's CCC combines Pearson's R (corresponding to precision) with a bias correction factor (corresponding to accuracy) capturing both how well the data fall on a straight line and how close that line is to the line of equality (*y* = *x*). Thus, CCC is lower if there's either random scatter (low precision) or systematic bias (lack of accuracy). We categorized the performance based on cutoffs of Lin's CCC proposed by McBride [70]: poor (< 0.90), moderate (0.90–0.95), substantial (0.95–0.99), and almost perfect (> 0.99) agreement. Additionally, we evaluated the quality‐of‐fit in the following subgroups: (1) WM ROIs versus GM ROIs and (2) left versus right hemisphere ROIs.

### 3.7 Comparison of Fixed‐T and Fixed‐N Regimes

As described in Equation (1), the frequency‐dependence in MK can be contributed to by structural disorder (through ${\bar{K}}_{\mathrm{var}}$) and water exchange (through h[q]). To investigate which term dominated the observed kurtosis frequency‐dependence in fixed‐T and fixed‐N regimes (Figure 1B), we calculated ${\bar{K}}_{\mathrm{var}}$ and h[q] using Equations (6) and (7), respectively, based on median values of each model parameter in global WM and cortical GM. We investigated the kurtosis frequency‐dependence within a peak frequency range of 23–89 Hz, the same range as the in vivo acquisition protocol (Table 1). Optimized $t_{M}$ for each gradient waveform was calculated in the same way as in vivo acquisition (Supporting Information Methods 1). For fixed‐T regime, gradient waveforms of 30 Hz‐N1, 65 Hz‐N2, and 90 Hz‐N3 were used with T fixed at 80 ms. For visualization, simulated MK at frequencies from 30 to 90 Hz were nonlinearly (spline) interpolated with step size of 1 Hz. For fixed‐N regime, N=1 was used across all frequency range from 23 to 89 Hz with a step size of 1 Hz.

### 3.8 Finding the Best Combination of Gradient Waveforms

To determine the best combination of gradient waveforms for estimating each model parameter, we used the Cramér–Rao Lower Bound (CRLB) method [71, 72]. We fixed $K_{\infty }$ as this parameter is not related to frequency‐dependence. CRLBs of four other parameters were calculated to find the optimal combination of waveforms. Given the number of parameters P and the number of gradient waveforms M, the log‐likelihood for an observed y can be derived using the parameter vector $\theta \in {\mathbb{R}}^{P}$ and the forward model (Equations 1, 6, and 7) $h\left(\theta \right)\in {\mathbb{R}}^{M}$:

(9) $$\ell \left(\theta \right)=-\frac{M}{2}\log\left(2\pi \sigma^{2}\right)-\frac{1}{2\sigma^{2}}{\left\|y-h\left(\theta \right)\right\|}^{2},$$

where $\sigma^{2}$ is the variance of noise assumed to be i.i.d Gaussian. Given the Jacobian matrix $J_{i}\left(\theta \right)=\partial h\left(\theta \right)/\partial \theta_{i}$, the Fisher information matrix $I_{i,j}$ is the expected outer product of the first derivative of ℓ(θ):

(10) $$I_{i,j}=E\left[\frac{\partial^{2}\ell }{\partial \theta_{i}\partial \theta_{j}}\right]=\frac{1}{\sigma^{2}}\;J_{i}^{T}J_{j}.$$

CRLB states that for any unbiased estimator $\hat{\theta }$, covariance matrix $\mathrm{Cov}\left(\hat{\theta }\right)\geq I{\left(\theta \right)}^{-1}$. Thus, the diagonal elements of $I^{-1}$ give lower bounds ($\mathrm{CRL}B_{i}$) on the variance of each unbiased parameter estimates $\theta_{i,\text{true}}$. As the scales of the parameters are all different (e.g., $t_{\mathrm{ex}}\in$ [1, 200] ms and $f_{\mathrm{ic}}\in$ [0.01, 0.99]), we computed the coefficient of variation $cv_{i}=\sqrt{\mathrm{CRL}B_{i}}/|\theta_{i,\text{true}}|$ for each parameter $\theta_{i}$. Model estimated parameters from the ROI analysis were used as $\theta_{i,\text{true}}$. For each ROI, the top 10 combinations were weighted by the opposite of the rank (i.e., rank 1 is weighted by 10 and rank 10 is weighted by 1); each combination obtained an accumulated score based on the sum of the weights. All 135 cortical, subcortical GM, and WM ROIs were utilized.

## 4 Results

### 4.1 Frequency Dependence of Mean Diffusivity and Kurtosis in Global WM and Cortex

**FIGURE 3:**
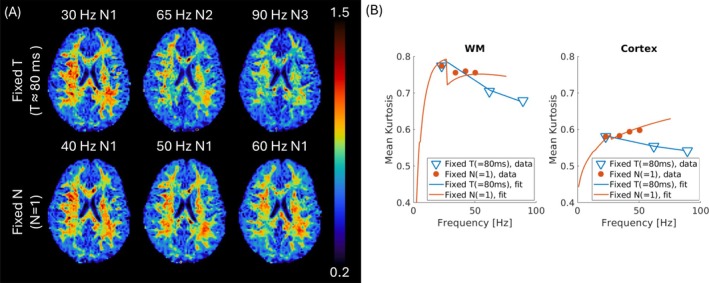
(A) Mean kurtosis (MK) maps in axial view at each input frequency from a single subject in the fixed‐T regime (top row) and fixed‐N regime (bottom row). MK values ranged from 0.2 to 1.5. (B) Both measured and fitted MK show that in the fixed‐T regime, MK decreased with frequency in both white and gray matter (WM and GM); in the fixed‐N regime, MK increased with frequency in GM, whereas MK showed weak frequency dependence in WM. The median MK values within global WM and cortical GM in fixed‐T (blue triangles) and fixed‐N (orange‐filled circles) regimes showed trends consistent with the qualitative assessment. The data points of all median MK values are placed at the peak frequency based on the corresponding power spectra. Solid lines represented fitting results from parameter estimation.

The MK showed clear and distinct frequency‐dependence between regimes (Figure 3). Blue triangles are fixed‐T regime data points located at corresponding peak frequencies of 23.2, 62.5, and 88.3 Hz (Table 1), showing decreased MK with frequency in both WM and cortex. Orange‐filled circles are fixed‐N regime data points located at corresponding peak frequencies of 23.2, 34.0, 42.2, and 51.6 Hz (Table 1), showing monotonic increased MK with frequency in cortex and weak (or irregular) frequency dependence of MK in WM. Fitting results generally followed the trend of the measured MK. In the fixed‐N regime, however, we observed an abrupt drop of measured MK after the first data point (30 Hz‐N1), especially in WM (Figure 3B, orange lines). This is due to the use of a nonideal $t_{M}$ (7.4 ms) in 30 Hz‐N1 waveform, whose optimized $t_{M}$ (41.7 ms) is too long to be used in practice (given TE = 118 ms).

Across all subjects, the same trend was observed: in global WM, dependence of MK was weak with irregular trend; however, in cortical GM, MK monotonically increased with frequency (Figure S2A). In comparison, the frequency dependence of mean diffusivity was subtle (Figure S3A). ROI analysis in global WM and cortical GM confirmed the expected monotonic increase of MD with frequency in both fixed‐T and fixed‐N regimes, with deviations less than 1% across subjects (Figures S2B and S3B).

Given the weak frequency‐dependence of MK in WM, we further investigated the potential influence of baseline fluctuations, such as eddy current or mechanical vibration. From the distilled water phantom (inner bottle) surrounded with ice (outer bottle) (Figure S1A), median values of MK across the ROI (Figure S1B) were almost 0 (< 10^−5^) with no noticeable frequency‐dependence (Figure S1C). The results show that the frequency‐dependence of MK is not related to potential external causes of temporal change in MK.

### 4.2 Regional Variation of Microstructural Parameters

**FIGURE 4:**
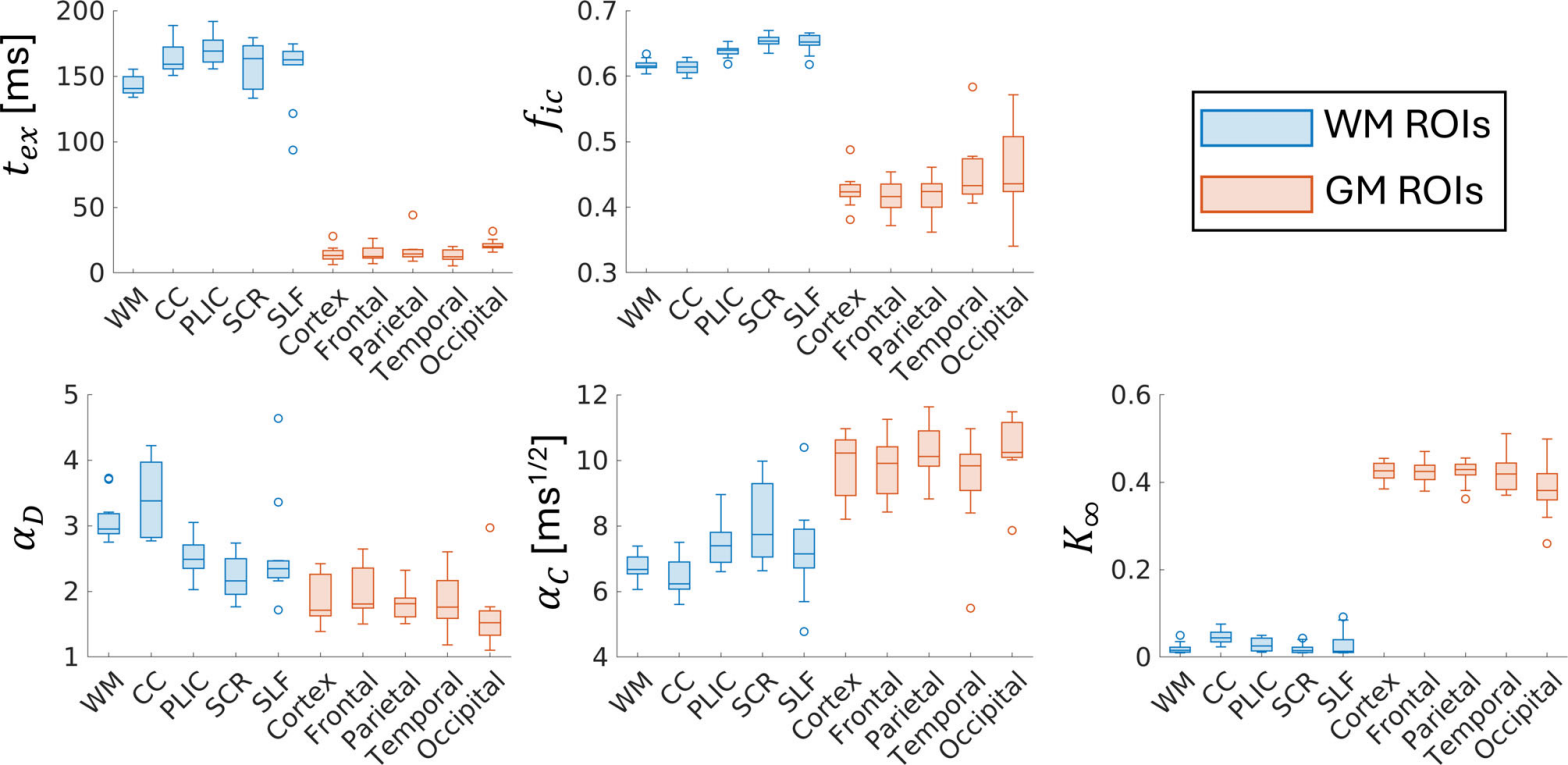
Estimated model parameters, including water exchange time ($t_{\mathrm{ex}}$), intracellular signal fraction ($f_{\mathrm{ic}}$), extracellular tortuosity ($\alpha_{D}$), frequency‐dependence factor ($\alpha_{C}$), and asymptotic kurtosis value in the long‐time limit ($K_{\infty }$) shown as boxplots across all 11 subjects for five white matter (WM) regions (blue) and gray matter (GM) regions (orange). WM ROIs included the corpus callosum (CC; genu, body, splenium combined); posterior limb of internal capsule (PLIC); superior corona radiata (SCR); and superior longitudinal fasciculus (SLF). For PLIC, SCR, and SLF, the left and right hemispheres were combined for each ROI. GM ROIs were combined into frontal, parietal, temporal, and occipital lobes, with hemispheres merged. The box indicates the first and third quartiles within each region, and the horizontal line indicates the median. Whiskers are 1.5 times the interquartile range.

We fitted the adiabatic Kärger model (Equations 1, 6, and 7) to frequency‐dependent MK across the 48 WM and 70 GM ROIs for all 11 subjects and estimated $t_{\mathrm{ex}}$, $f_{\mathrm{ic}}$, $\alpha_{D}$, $\alpha_{C}$, and $K_{\infty }$. Representative WM ROIs, including the corpus callosum (CC), posterior limb of internal capsule (PLIC), superior corona radiata (SCR), and superior longitudinal fasciculus (SLF), and lobar GM ROIs (frontal, parietal, temporal, and occipital) revealed systematic differences (Figure 4, Table S1). WM showed longer $t_{\mathrm{ex}}$, higher $f_{\mathrm{ic}}$, and higher $\alpha_{D}$ compared to GM, consistent with dense, myelinated axons and anisotropic extracellular space. In contrast, GM exhibited shorter $t_{\mathrm{ex}}$ and higher $K_{\infty }$, reflecting greater tissue heterogeneity and partial volume effects with CSF. Among the four WM ROIs, the longest $t_{\mathrm{ex}}$ was observed in PLIC (164.14 ms). Among GM ROIs, the longest $t_{\mathrm{ex}}$ was observed in the occipital lobe (20.43 ms), followed by the parietal and frontal lobes, with the temporal lobe showing the shortest $t_{\mathrm{ex}}$. Among all ROIs, $f_{\mathrm{ic}}$ was comparable among all GM ROIs, with the occipital lobe showing the largest inter‐subject variability as evaluated by the interquartile range. Among WM ROIs, the highest and lowest $\alpha_{D}$ were found in CC and SCR, respectively. Among GM ROIs, the highest and lowest $\alpha_{D}$ were found in the parietal lobe and occipital lobe, respectively. $\alpha_{C}$ was generally larger in GM ROIs compared to WM ROIs. Across all ROIs, the highest $\alpha_{C}$ was observed in occipital lobe (10.27) and the lowest $\alpha_{C}$ was found in CC (6.34). Aligning with $\alpha_{D}$, the highest $K_{\infty }$ across WM ROIs was found in CC, and the lowest $K_{\infty }$ across GM ROIs was found in occipital lobe.

**FIGURE 5:**
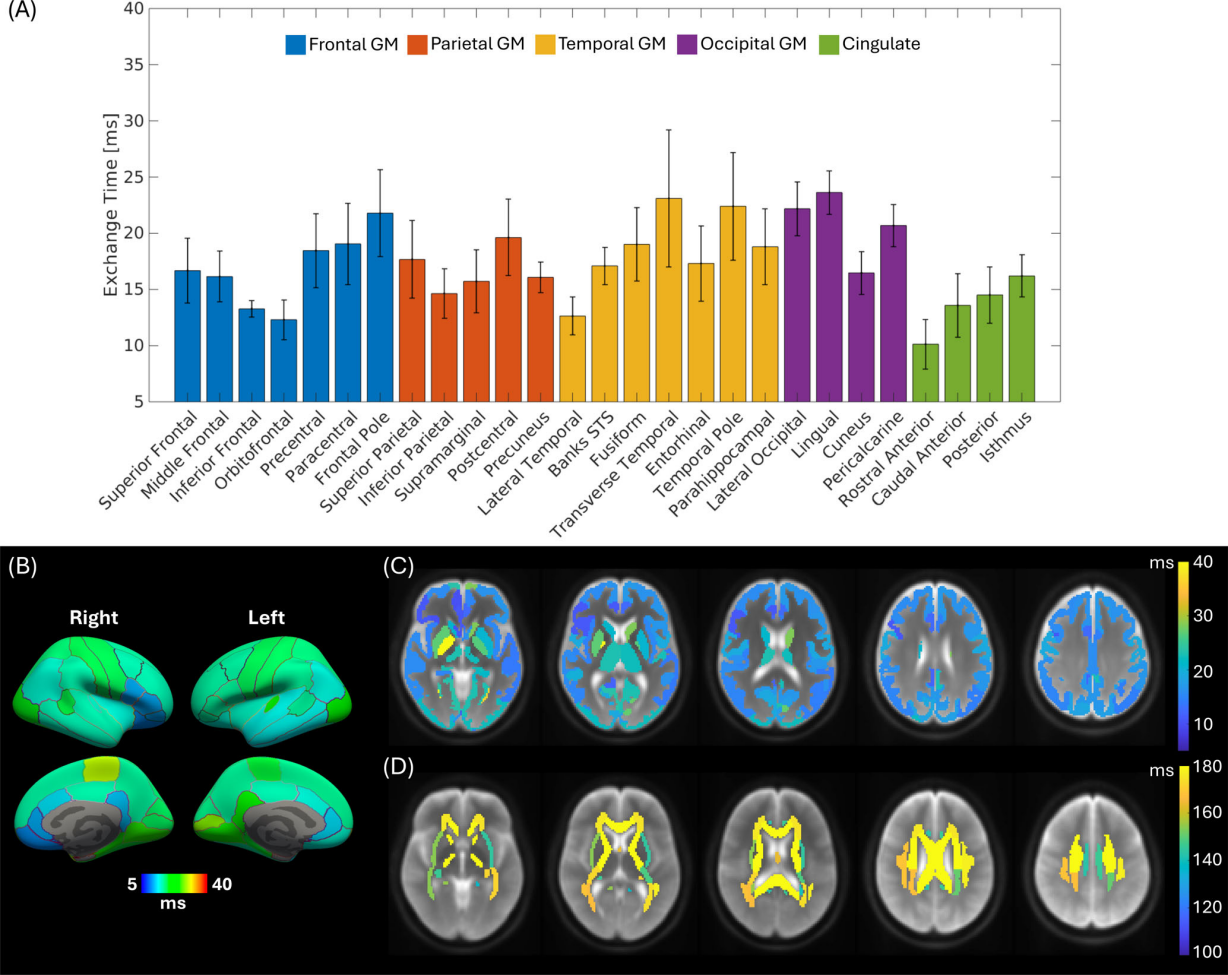
Cortical and subcortical exchange time estimated by multi‐parametric model fitting to mean kurtosis (MK). (A) Bar plots showing mean and standard error bars across all subjects for each cortical region of interest (ROI), colored differently for each lobe. For the bar plots, the adiabatic Kärger model was fitted to the median MK values across all voxels within both the left and right hemispheres of each ROI, yielding a roughly averaged value for the two sides. (B) Cortical surface projection of the exchange time in 28 cortical ROIs displayed a range from 5 to 40 ms. The water exchange times in (C) gray matter ROIs (cortex + subnuclei) and (D) white matter ROIs were overlaid on five axial slices of *T*_2_‐weighted images in MNI space.

Mapping water exchange time $t_{\mathrm{ex}}$ across cortical and subcortical ROIs revealed biologically meaningful spatial gradients (Figure 5). Cortical projection (Figure 5A) and bar graphs (Figure 5B) of $t_{\mathrm{ex}}$ provided structural patterns across the cortical ROIs. The shortest $t_{\mathrm{ex}}$ was observed in rostral anterior cingulate (6.11 ms). In addition, we found anterior‐to‐posterior spatial gradient of $t_{\mathrm{ex}}$ showing longer $t_{\mathrm{ex}}$ in the posterior cingulate (12.91 ms) compared to rostral (6.11 ms) or caudal anterior (7.81 ms) cingulate, compatible with known variations in myelination. The longest $t_{\mathrm{ex}}$ among cortical ROIs was found in lingual cortex (21.37 ms). Expanding the search range from cortical ROIs to all GM ROIs, including subnuclei, revealed that the pallidum, a region associated with motor control, motivation, and cognition [73], had the longest $t_{\mathrm{ex}}$ (32.87 ms). Among all 27 WM ROIs (combining left and right ROIs), anterior limb of internal capsule (ALIC) showed the longest $t_{\mathrm{ex}}$ (168.00 ms), approximately 4 ms longer than PLIC, with both regions having among the longest $t_{\mathrm{ex}}$, aligning with their roles as heavily myelinated projection pathways. Figure 5C,D summarizes the spatial gradient of $t_{\mathrm{ex}}$ in GM and WM ROIs. These results highlight the sensitivity of OGSE‐based exchange mapping to regional differences in membrane permeability.

### 4.3 Assessment of Mean Kurtosis Prediction Quality

Across all 7788 data points (11 subjects, 6 gradient waveforms, and 118 ROIs), model fits showed good agreement with measured MK (*R* = 0.98, Lin's CCC = 0.98, RMSE = 0.02; Figure S4A). Based on McBride's criteria for categorization of the results, Lin's CCC [70], model‐estimated MKs were in substantial agreement (0.95 ≤ Lin's CCC ≤ 0.99) for all data points. In the Bland–Altman plots showing the residuals (the difference between fitted and measured MK) versus their mean (Figure S4B), most ROIs fell within the limit of agreement (mean ± 1.96 × standard deviation). When comparing subsets of the data (left ROIs vs. right ROIs and GM ROIs vs. WM ROIs), the prediction quality was comparable between the groups (Figure S4C,D) with a bias of 0.00 and an RMSE < 0.05. The left and right ROIs showed substantial agreement (0.95 ≤ Lin's CCC ≤ 0.99), while the GM and WM ROIs showed moderate agreement (0.90 ≤ Lin's CCC < 0.95).

### 4.4 Comparison of Fixed‐T and Fixed‐N Regimes

**FIGURE 6:**
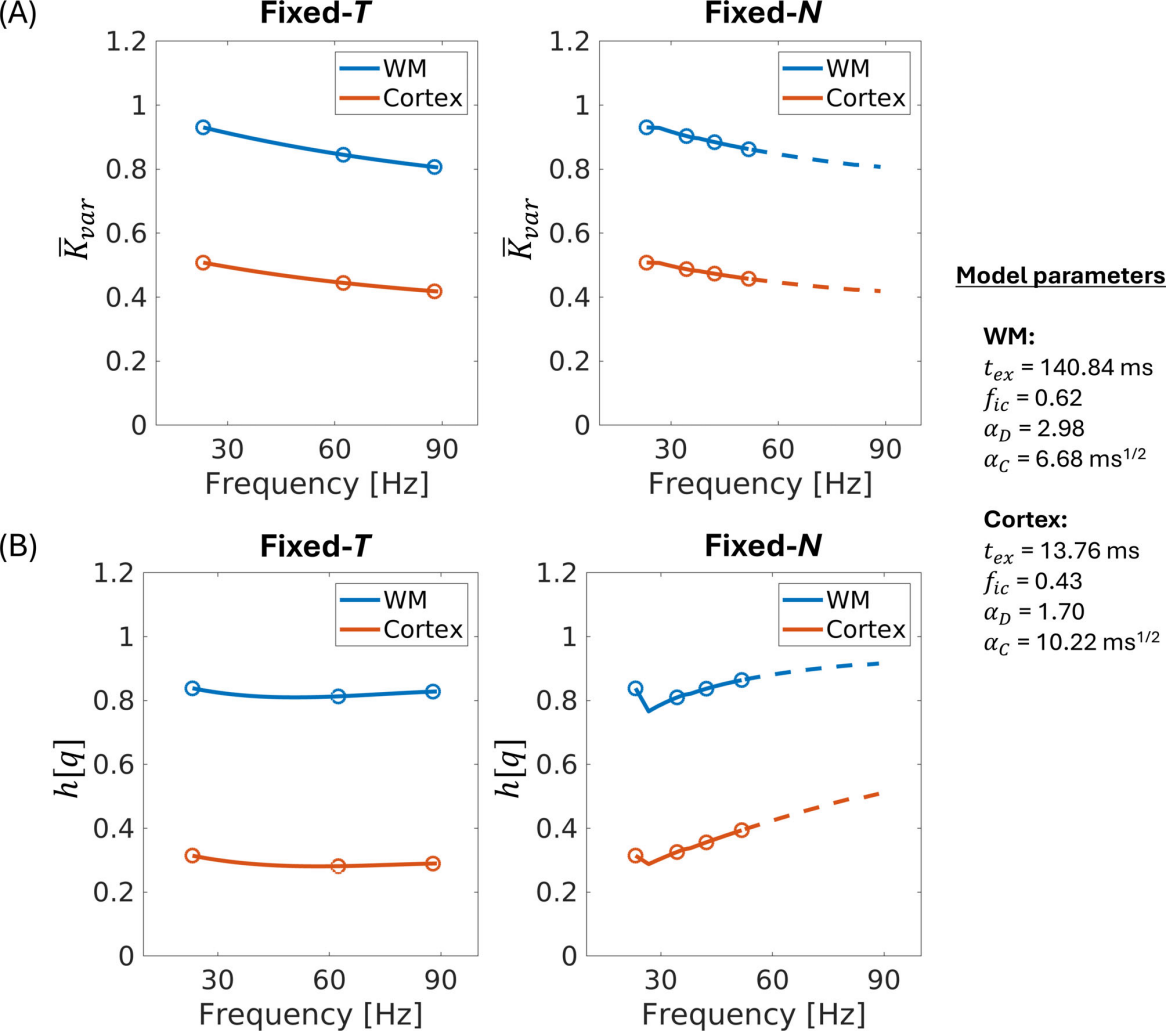
(A) ${\bar{K}}_{\mathrm{var}}$ was calculated based on Equation (6) and the median values of $f_{\mathrm{ic}}$, $\alpha_{D}$, and $\alpha_{C}$ estimated in white matter (WM, blue lines) and cortical gray matter (GM, orange lines) for fixed‐T and fixed‐N regimes. Frequency dependence of ${\bar{K}}_{\mathrm{var}}$ was similar in both regimes, showing monotonic decrease of ${\bar{K}}_{\mathrm{var}}$ with frequency. For fixed‐N regime, data points with peak frequency > 52 Hz were projected in dashed lines, as they are out of range from the actual in vivo acquisition protocol. (B) h[q] was calculated based on Equation (7) and the median values of $t_{\mathrm{ex}}$ estimated in WM (blue lines) and cortical GM (orange lines) for fixed‐T and fixed‐N regimes. Circles overlaid on each fitted line approximately indicate peak frequencies of actual in vivo acquisition. Relatively weak frequency dependence of h[q] was observed in fixed‐T regime, whereas monotonic increase of h[q] with frequency was observed in fixed‐N regime. The peak frequency of power spectrum of OGSE waveform used in our experiment was indicated by the data points.

To disentangle the contributions of structural disorder and exchange, we investigated the frequency dependence of ${\bar{K}}_{\mathrm{var}}$ (Equation 6) and h[q] (Equation 7) in the fixed‐T and fixed‐N regimes, respectively, using the median values of model parameters within global WM ($t_{\mathrm{ex}}$ = 140.84 ms, $f_{\mathrm{ic}}$ = 0.62, $\alpha_{D}$ = 2.98, and $\alpha_{C}$ = 6.68 ms^1/2^) and cortical GM ($t_{\mathrm{ex}}$ = 13.76 ms, $f_{\mathrm{ic}}$ = 0.43, $\alpha_{D}$=1.70, and $\alpha_{C}$ = 10.22 ms^1/2^). In both global WM and cortical GM, ${\bar{K}}_{\mathrm{var}}$ decreased monotonically with frequency in both regimes (Figure 6A); in contrast, a very weak frequency‐dependence of h[q] was observed in the fixed‐T regime, and a monotonic increase of h[q] with frequency was observed in the fixed‐N regime (Figure 6B). For the fixed‐N regime, data points acquired with a gradient waveform of peak frequency > 52 Hz are displayed in dashed lines to match the frequency range of the fixed‐N regime for in vivo acquisition (23–52 Hz). Color‐matched circles are overlaid on each fitted line, representing approximate peak frequencies of six data points (23, 34, 42, 52, 63, and 88 Hz) acquired in vivo. For those gradient waveforms with optimized $t_{M}$ < 7.4 ms or > 40 ms, their mixing times were set at the scanner‐allowed minimum $t_{M}$ of 7.4 ms, leading to a discontinuity of fitted MK around 30 Hz. The main factor driving frequency‐dependence in the fixed‐T regime was ${\bar{K}}_{\mathrm{var}}$ related to structural disorder, whereas the main factor driving frequency‐dependence in the fixed‐N regime was h[q] related to water exchange between compartments.

### 4.5 Optimal Combinations of Gradient Waveforms

Using all gradient waveforms always showed the best performance in terms of coefficient of variance cv. Thus, we focused on finding the best combination of four gradient waveforms, which is the minimum number of waveforms to avoid the degeneracy problem. The best combination of four gradient waveforms was determined for all model parameters except the fixed parameter $K_{\infty }$. The best waveform combination for estimating $t_{\mathrm{ex}}$, $f_{\mathrm{ic}}$, $\alpha_{D}$, and $\alpha_{C}$ were [30 Hz‐N1, 50 Hz‐N1, 60 Hz‐N1, 90 Hz‐N3], [30 Hz‐N1, 40 Hz‐N1, 60 Hz‐N1, 90 Hz‐N3], [30 Hz‐N1, 40 Hz‐N1, 50 Hz‐N1, 60 Hz‐N1], and [30 Hz‐N1, 40 Hz‐N1, 60 Hz‐N1, 90 Hz‐N3], respectively. 30 Hz‐N1 was a gradient waveform necessary to include, and 90 Hz‐N3 was the second most important waveform as three model parameters needed it for the best estimation. Median values of cv of the optimized combination across all ROIs for each parameter ($t_{\mathrm{ex}}$, $f_{\mathrm{ic}}$, $\alpha_{D}$, and $\alpha_{C}$) were 0.71, 0.34, 1.29, and 1.23, respectively. Accumulated scores and median values of cv across all ROIs of all four gradient‐waveform combinations for all four parameters are summarized in Table S2.

## 5 Discussion

In this study, we demonstrated that the discrepancy of kurtosis frequency‐dependence between fixed‐T and fixed‐N regimes can be explained mechanistically using the adiabatic Kärger model incorporating both structural disorder [8] and inter‐compartmental water exchange [30, 37]. We considered compartmental anisotropy not just in the intracellular space but also in the extracellular space. WM exhibited longer water exchange times $t_{\mathrm{ex}}$, higher intracellular signal fractions $f_{\mathrm{ic}}$, and greater extracellular tortuosity $\alpha_{D}$ than cortical GM, reflecting the restricted permeability of the myelin sheath, densely packed axons, and anisotropic extracellular environment. Conversely, GM showed a larger frequency‐dependent factor $\alpha_{C}$ and higher asymptotic kurtosis $K_{\infty }$, consistent with its more heterogenous microstructure and partial volume effects with CSF. Together, these findings support the complementary sensitivity of the two OGSE regimes and highlight their utility for probing distinct aspects of tissue micro‐geometry in vivo.

### 5.1 Estimation of Inter‐Compartmental Water Exchange Time

Inter‐compartmental water exchange is a central process underlying the time‐ and frequency‐dependence of kurtosis. The ability to probe exchange in the living human brain has been enabled by advances in gradient hardware that exceed the ˜80 mT/m limits of conventional clinical scanners. Using the first‐generation Connectome MRI scanner equipped with 300 mT/m gradients, Uhl et al. reported exchange times of 30–40 ms with pulse‐gradient‐based Neurite Exchange Imaging [74]. Chakwizira et al. extended this work using free‐gradient waveforms, showing that WM exchange times are at least twice as long as those in GM, both with standard clinical gradients (80 mT/m) and ultra‐strong gradient systems (*G*_max_ = 200–300 mT/m on the 3 T MAGNUS scanner) [75]. The highest‐performing system to date, Connectome 2.0, achieves up to 500 mT/m gradient strength and 600 T/m/s slew rate [38, 39]. Using this platform, we recently demonstrated improved estimation of apparent exchange times in GM, particularly for short exchange times (< 20 ms), with pulsed‐gradient sequences at three diffusion times (13–30 ms) [68]. In the present study, we build on that work by extending the feasibility of exchange measurements on Connectome 2.0 from pulsed‐gradient to oscillating‐gradient waveforms, enabling sensitive probing of short exchange times (< 20 ms). This establishes OGSE as a complementary tool for probing fast exchange dynamics that are inaccessible with pulsed‐gradient spin‐echo (PGSE).

### 5.2 Spatial Variations of Tissue Parameters

Our regional analyses further showed that OGSE‐derived parameters reflect the underlying tissue composition and organization. For example, $t_{\mathrm{ex}}$ were longest in highly myelinated WM tracts such as the ALIC and PLIC, a major part of the descending pathway for motor control [76], consistent with restricted permeability imposed by compact axons with robust myelin sheaths [77, 78]. Similarly, cortical regions with relatively high myelin content, including the visual, motor, and somatosensory cortices as well as the posterior cingulate, also exhibited longer $t_{\mathrm{ex}}$, in line with prior MRI studies [68, 74, 79]. Within the cingulate gyrus, we observed an anterior‐to‐posterior gradient of increasing $t_{\mathrm{ex}}$, mirroring known variations in cortical myelination [80, 81].

$f_{\mathrm{ic}}$ varied most in the occipital lobe, suggesting individual differences in neuronal density. This observation parallels histological reports of variability in visual cortex microstructure [82] and functional and retinotopic variability across individuals in early visual areas (e.g., V2 and V3) [83]. $\alpha_{D}$ was highest in the corpus callosum, reflecting its dense, coherently aligned axons with high anisotropy in the extracellular space, while the lowest values of extracellular tortuosity in the superior corona radiata are consistent with the known fan‐shaped, highly dispersed axons, that is, low anisotropy, in this region [84, 85]. Among GM ROIs, the occipital lobe showed both high $f_{\mathrm{ic}}$ and low $\alpha_{D}$, consistent with prior evidence of densely packed but highly neuritic in this region [86]. Together, these spatial patterns highlight how the adiabatic Kärger model applied to OGSE provides biologically interpretable metrics, linking MR signal changes to microstructural features such as membrane integrity ($t_{\mathrm{ex}}$), neurite density ($f_{\mathrm{ic}}$), extracellular anisotropy ($\alpha_{D}$), and tissue heterogeneity ($K_{\infty }$).

### 5.3 Temporal Sensitivity of Two Frequency‐Varying Regimes

Our results demonstrate that the frequency‐dependence of kurtosis depends strongly on how oscillating frequency is modulated, whether by varying T or N. A persistent challenge in OGSE is defining the effective diffusion time $\left(\Delta_{\mathrm{eff}}\right)$, since it is model‐specific [7]. For an ideal cosine‐based oscillating‐gradient waveform, we previously defined an $\Delta_{\mathrm{eff}}$ in the slow exchange limit $\left(t_{\mathrm{ex}}/T\ll 1\right)$ for both fixed‐T and fixed‐N regimes [12]. The same formulation can be used to get an approximate sense of $\Delta_{\mathrm{eff}}$ in a trapezoid‐cosine gradient waveform as follows:

(11) $$\Delta_{\mathrm{eff}}=\left\{\begin{array}{l} T\left(1-\frac{15}{4}\frac{1}{\omega_{p}^{2}T^{2}}\right),\;\left(\text{fixed}-T\right)\\ \frac{2\pi N}{\omega_{p}}\left(1-\frac{15}{16\pi^{2}}\frac{1}{N^{2}}\right),\;\left(\text{fixed}-N\right) \end{array}\right.$$

where $\omega_{p}$ is the peak frequency from power spectrum ${\left|q_{\omega }\right|}^{2}$, N is the number of oscillating cycles, and T is the duration of diffusion encoding block ($T=2\pi N/\omega_{0}+t_{r}$, where $\omega_{0}$ is the input frequency, and $t_{r}$ is ramp time). Here, we assume a negligible mixing time $\left(t_{M}\ll T\right)$ for simplicity.

In the fixed‐T regime, $\Delta_{\mathrm{eff}}$ remains close to the T with increasing frequency (Figure S5A), but the encoding spectrum shifts toward higher frequencies. This explains why fixed‐T acquisitions primarily capture structural disorder effects associated with diffusion coarse‐graining at longer time scales (Section 4.4). In contrast, in the fixed‐N regime, $\Delta_{\mathrm{eff}}∼2\pi /\omega_{p}$ decreases with frequency (Figure S5B), offering a broader temporal window for detecting inter‐compartmental exchange (Section 4.4).

Together, these findings emphasize that frequency dependence in OGSE cannot be interpreted solely through $\Delta_{\mathrm{eff}}$, but must also account for the spectral weighting imposed by the gradient waveform. Fixed‐T waveforms emphasize sensitivity to the underlying diffusion spectrum at longer times, whereas fixed‐N waveforms enhance sensitivity to membrane integrity and water exchange processes across a wider temporal range. This distinction has direct implications for experimental design, as it determines whether OGSE measurements preferentially probe inter‐compartmental exchange dynamics or the spectral distribution of restricted and hindered motion. Hao et al. reported the biphasic frequency‐dependence of MK from their mixing‐time‐minimized OGSE waveform, especially in the subcortical nuclei regions, and the MK increase with effective frequency from the PGSE waveform [15]. Combining with our findings, this study shows the need for rigorous sensitivity analysis with various gradient waveforms, not just tied into T and N, but also $t_{M}$, types of waveform (sine vs. cosine), and encoding dimension (i.e., linear, planar, and spherical) in the future.

### 5.4 Optimized Parameter Estimation Using Different Waveform Combinations

We have investigated the optimal combination of gradient waveforms, especially in the case of utilizing four gradient waveforms, the minimum number of gradient waveforms to avoid the degeneracy problem with fixed $K_{\infty }$. The best combination for $t_{\mathrm{ex}}$ was [30 Hz‐N1, 50 Hz‐N1, 60 Hz‐N1, 90 Hz‐N3], which is leaning toward fixed‐N than fixed‐T regime. Besides, the best combination for extracellular tortuosity ($\alpha_{D}$) was a full set of fixed‐N regime [30 Hz‐N1, 40 Hz‐N1, 50 Hz‐N1, and 60 Hz‐N1]. For $f_{\mathrm{ic}}$, on the other hand, the lowest cv was observed in the combination of [30 Hz‐N1, 40 Hz‐N1, 60 Hz‐N1, and 90 Hz‐N3]. Given T of 40 Hz‐N1 is 84.1 ms, close to those of 30 Hz‐N1 (77.3 ms) and 90 Hz‐N3 (80.0 ms), this gradient waveform combination can also be considered as an alternative fixed‐T regime. These observations are consistent with the results from comparison of fixed‐T and fixed‐N regimes disentangling the contributions of structural disorder and exchange (Sections 4.4 and 5.3). As T and N are not the only factors that modify the gradient waveform, additional variables such as the $t_{M}$ and phase of the waveform (cosine‐OGSE, sine‐OGSE, or their superposition) may need to be considered. Incorporating a wider range of frequency spectra in future studies may further improve OGSE protocol optimization.

### 5.5 Advantages of Ultra‐High Gradient MR System

In this study, we demonstrated the feasibility of acquiring in vivo human brain dMRI acquisition with OGSE at frequencies up to $\omega_{\max}/2\pi$ = 90 Hz and *b* values up to $b_{\max}$ = 2 ms/μm^2^ ($b_{\max}\times \omega_{\max}/2\pi$ = 0.18 μm^−2^) on the Connectome 2.0 scanner, without safety issues or noticeable peripheral nerve stimulation. Previous in vivo OGSE scans have relied on high‐performance gradient systems such as the GE 3 T MAGNUS head‐only system [23, 87], the Phillips 3 T Achieva with an insert gradient coil [15, 88], and the GE 3 T MAGNUS 2.0 head‐only system [75, 89] with maximum gradient strengths of 200, 200, and 300 mT/m, respectively. The highest OGSE performance achieved in previous studies was $b_{\max}\times \omega_{\max}/2\pi$ = 0.14 μm^−2^ with $b_{\max}$ = 4 ms/μm^2^ and $\omega_{\max}/2\pi$ = 35 Hz [75], with other in vivo human studies achieving even lower values of $b_{\max}\times \omega_{\max}/2\pi$ [23, 27, 28]. Although such high‐performance acquisitions extend beyond the capabilities of standard clinical systems and thus present challenges for translation, they provide critical insights into brain tissue microstructure and guide protocol optimization. These results underscore the importance of ultra‐strong gradient platforms not only for advancing scientific discovery but also for informing future translational applications.

### 5.6 Outlook

This work has several limitations that suggest directions for future research. First, our analysis was performed at the ROI level, which may mask spatial heterogeneity. Extending the Kärger framework to voxel‐wise model fitting could improve microstructural specificity across regions. Second, the 2‐mm isotropic resolution introduces partial volume effects that may confound parameter estimates. Higher spatial resolution will be important to mitigate this issue. Third, we focused on linear‐tensor‐encoding cosine‐OGSE waveforms (Figure 1A). While we did not include PGSE or sine‐OGSE to achieve a sharp low‐frequency peak in the power spectrum at relatively high *b* values [27], we acknowledge that PGSE or sine‐OGSE may provide additional information about tissue microstructure [7]. The signal sensitivities of PGSE, sine‐OGSE, and cosine‐OGSE waveforms to tissue microstructure will be compared in future studies. Furthermore, more advanced encoding schemes, such as b‐tensor encoding, may better disentangle inter‐compartmental exchange from compartmental diffusion [75]. Finally, for model tractability, we considered only neurites in the intracellular space, combining soma and extracellular matrix into the extracellular space and collecting kurtosis contributions from all other non‐exchanging compartments into a single $K_{\infty }$ value. Future work could pursue more granular compartmental separation, for example, by adapting methods such as Soma and Neurite Density Imaging with Exchange [90] for OGSE. Together, these limitations highlight the importance of developing higher‐resolution, voxel‐wise, and multi‐compartment‐sensitive OGSE frameworks to fully capture the complexity of brain tissue microstructure.

## 6 Conclusion

In this study, we applied oscillating‐gradient waveforms in two frequency‐varying regimes (fixed‐T vs. fixed‐N) and demonstrated distinct patterns of kurtosis frequency‐dependence in the human brain. These differences can be explained by the combined effects of structural disorder and inter‐compartmental water exchange. In the fixed‐T regime, the MK frequency‐dependence is predominantly influenced by the structural disorder‐related term (${\bar{K}}_{\mathrm{var}}$) in comparison to the inter‐compartmental water exchange related term (h[q]), resulting in monotonic decline of MK with increasing frequency in both WM and GM. In the fixed‐N regime, on the other hand, the impact of h[q] is more pronounced than in the fixed‐T regime, leading to a confluence of structural disorder and inter‐compartmental water exchange characterized by their mixed frequency dependence. Leveraging the ultra‐strong gradients of the Connectome 2.0 scanner, we extended OGSE acquisitions to higher frequencies and showed that the fixed‐N regime is particularly sensitive for estimating exchange time. ROI‐based analyses revealed biologically meaningful spatial variations in membrane integrity, neurite density, extracellular anisotropy, and tissue heterogeneity. Together, these findings establish the feasibility of OGSE‐based exchange mapping in vivo and lay the foundation for future voxel‐wise, multi‐compartment analyses using the adiabatic Kärger model.

## Funding

This work was supported by ZonMw (04520232330012) and the National Institutes of Health (DP5OD031854, P41EB015896, P41EB030006, R01NS118187, R21AG085795, U01EB026996, U24NS137077).

## Supporting information


**Data S1:** Supporting Information.

## Data Availability

The source code for diffusion MRI preprocessing is shared in https://github.com/Connectome20, and GACELLE toolbox used for model fitting is shared in https://github.com/kschan0214/gacelle. Raw MR data are available from the corresponding author on reasonable request and after a data sharing agreement is executed between institutions.
