# Supplementary material for: Complementary Sensitivity of Fixed‐Time and Fixed‐Oscillation Regimes to Exchange and Structural Disorder in the Human Brain Revealed Using Oscillating‐Gradient Diffusion MRI With Ultra‐Strong Gradients
Source: Magn Reson Med. 2026 Feb 15;95(6):3429–44. doi: 10.1002/mrm.70300 (PMC13049281; doi:10.1002/mrm.70300)
Supplement: Supplementary file 1 — Data S1: Supporting Information. [file MRM-95-3429-s001.docx]

**Supporting Information**

**Complementary sensitivity of fixed-time and fixed-oscillation regimes to exchange and structural disorder in the human brain revealed using oscillating-gradient diffusion MRI with ultra-strong gradients**

Dongsuk Sung^1,2*^, Kwok-Shing Chan^1,2^, Julianna Gerold^1^, Wen Zhong^3^, Jialan Zheng^3^, Qiyuan Tian^3^, Hua Guo^3^, Susie Y Huang^1,2^, Hong-Hsi Lee^1,2^

^1^Athinoula A. Martinos Center for Biomedical Imaging, Department of Radiology, Massachusetts General, Charlestown, MA, United States

^2^Harvard Medical School, Boston, MA, United Sates

^3^School of Biomedical Engineering, Tsinghua University, Beijing, China

* Corresponding author:

Dongsuk Sung, PhD

Address: Athinoula A. Martinos Center for Biomedical Imaging, Department of Radiology, Massachusetts General Hospital, 149 13th Street, Room 2301, Charlestown, Massachusetts 02129, United States

E-mail : dsung2@mgh.harvard.edu

**This file includes:**

**Supporting Information Theory.
Supporting Information Methods 1.
Supporting Information Methods 2.
Supporting Information Figure S1.
Supporting Information Figure S2.**  **Supporting Information Figure S3.**  **Supporting Information Figure S4.
Supporting Information Figure S5.
Supporting Information Figure S6.
Supporting Information Figure S7.
Supporting Information Figure S8.
Supporting Information Table S1.
Supporting Information Table S2.**

**Supporting Information Theory. Frequency-dependent diffusivity due to structural disorders along neurites**

Due to structural disorders along neurites, such as randomly positioned axonal beadings or varicosities, the diffusivity frequency-dependence follows a power-law scaling^1-4^ with a dynamical exponent $\vartheta=(p+d)/2=1/2$, where the structural exponent $p$ is zero due to Poisson statistics (randomly positioned restrictions), and dimensionality $d$ is 1 along neurites. Based on the Equation A.7 in our previous study,^5^ the frequency($\omega$)-dependent dispersive diffusivity was given by

|  | $\mathcal{D}\left( \omega\right)=D_{\infty}+A\Gamma_{E}\left( 1-\vartheta\right)\left( -i\omega\right)^{\vartheta} ,$ | (S1) |
| --- | --- | --- |

where $D_{\infty}$ is the bulk diffusivity at long time, $A$ is the strength of restrictions, and $\Gamma_{E}$ is Euler’s Gamma function. With $\vartheta=1/2$ for randomly positioned restrictions along neurites, the **Equation (S1)** yielded

|  | $\mathcal{D}\left( \omega\right)=D_{\infty}+A\sqrt{\pi}\left( -i\omega\right)^{\frac{1}{2}}=\left( D_{\infty}+c\sqrt{\omega} \right)-i\cdot c\sqrt{\omega}$ | (S2) |
| --- | --- | --- |

with $c=A\sqrt{\pi/2}$. For a diffusion gradient waveform $G(t)$, the diffusion wave vector is defined as $q\left( t \right)=\int_{0}^{t} \gamma G\left( t^{'} \right) dt'$, and its Fourier transform $q_{\omega}$ defines the power spectrum of gradient waveform $\left| q_{\omega} \right|^{2}$. Based on the Gaussian phase approximation,^6,7^ the diffusion signal along neurites ($\hat{g}=\hat{n}$) up to the second order cumulant is

|  | $-\ln S\left( \hat{g}=\hat{n} \right) \simeq\int\frac{d\omega}{2\pi}\mathcal{D}\left( \omega\right)\left\vert q_{\omega} \right\vert^{2}=\int\frac{d\omega}{2\pi}\left( D_{\infty}^{\parallel}+c\sqrt{\vert\omega\vert} \right)\left\vert q_{\omega} \right\vert^{2}.$ | (S3) |
| --- | --- | --- |

Here we only consider the real part of dispersive diffusivity (**Equation (S2)**)**,** since its imaginary part is an odd function and does not contribute to the signal (**Equation (S3)**). Given that the apparent diffusivity is defined as $D\equiv-\frac{1}{b}\ln S$ with $b=\int\frac{d\omega}{2\pi}\left| q_{\omega} \right|^{2}$, we obtain the intra-cellular diffusivity along neurites

|  | $D_{ic}^{\parallel}\simeq D_{\infty}^{\parallel}+\frac{c}{b}\int\frac{d\omega}{2\pi}\sqrt{\vert\omega\vert}\left\vert q_{\omega} \right\vert^{2}.$ | (S4) |
| --- | --- | --- |

**Supporting Information Methods 1. Optimal mixing time acquisition**

As can be seen in **Figure 2**, the power spectrum of a trapezoidal cosine gradient waveform creates side lobes around the main peak frequency, and the main peak frequency is not the same as the target frequency we input in the sequence card. The side lobes and the peak frequency of the power spectrum alter by mixing time. Thus, we aimed to find the optimal mixing time that can maximize the ratio of “peak amplitude” and “side lobe amplitude” as well as minimize the difference between target frequency and actual peak frequency. Among these two objectives, we mainly focused on the first one, which is maximizing the “peak ratio” (**Figure S6A**), and as a side rule we wanted the left and right secondary side lobes next to the main lobe to be balanced. We have incremented mixing time from the minimum mixing time the scanner can implement (7.4 ms) to 50 ms with a step size of 0.1 ms for each gradient waveform and evaluated both peak ratio and absolute difference between peak and target frequency (**Figure S6B**). For 60Hz-N1, 65Hz-N2, and 90Hz-N3, the mixing time maximized the peak ratio was selected as the optimal mixing time. For 30Hz-N1, 40Hz-N1, and 50Hz-N1, the minimum scanner allowed mixing time (7.4 ms) resulted in the maximum peak ratio. However, the minimum mixing time (7.4 ms) was not the true optimal mixing time for these waveforms because true optimal mixing times were 1.8, 1.0, and 0.5 ms for 30Hz-N1, 40Hz-N1, and 50Hz-N1, respectively, when we further extended the mixing time range down to 0 ms. If we chose the optimal mixing time as 7.4 ms for these three waveforms, the side lobes were unbalanced between left and right sides of the main lobe. We found that the local maximum in the peak ratio vs. mixing time plot exhibited balanced side lobes in corresponding power spectrum. Thus, for 40Hz-N1 and 50Hz-N1, we chose the next peak which are mixing time of 31 and 24.7 ms. In the case of 30Hz-N1, however, the next optimal mixing time peak is located at 41.7 ms which is larger than the maximum limit of mixing time for TE/TR = 118/7600 ms. We decided to use the minimum mixing time of 7.4 ms for 30Hz-N1 rather than increasing TE, as we did not want to sacrifice the signal-to-noise ratio over optimal mixing time for just one gradient waveform.

We also compared our optimal mixing times with the theoretically derived optimal mixing time described in a previous literature.^8^ Based on the previous study, to maximize the peak amplitude of power spectrum at the main lobe peak frequency ($\omega_{p}/2\pi$), the optimal mixing time ($t_{M}$) for a finite cosine OGSE waveform should satisfy the following equation.

|  | $\omega_{p}\left( \frac{2\pi N}{\omega_{i}}+t_{r}+t_{M} \right)=2\pi M, \left( M=1, 2, 3, 4, \ldots\right),$ | (S5) |
| --- | --- | --- |

where $\omega_{p}/2\pi$ is an input frequency (or targe frequency set in the sequence card), $t_{r}$ is a ramp time, and $N$ is the number of oscillations before the refocusing pulse. From **Equation S5**, we can get

|  | $t_{M}=\frac{2\pi M}{\omega_{p}}-\frac{2\pi N}{\omega_{i}}-t_{r}, \left( M=1, 2, 3, 4, \ldots\right).$ | (S6) |
| --- | --- | --- |

Compared to the optimal mixing time acquired by maximizing the ratio of peak amplitude and side lobe amplitude, the theoretically derived optimal mixing time (**Equation S6**) were almost the same with a difference of ~2$t_{r}$/$N$.

**Supporting Information Methods 2. OGSE frequency selection and the impact of optimized mixing time**

The selection of frequency was determined based on several factors including b-value constraints (maximal b-value $b_{max}=2$ ms/µm^2^), TE, maximum gradient strength and slew rate (*G*_max_ and *SR*_max_), and total waveform time $T$ determined by optimized mixing time $t_{M}$. To achieve $b_{max}=2$ ms/µm^2^ using the trapezoidal cosine waveform design (**Figure 1A**) with *G*_max_=500 mT/m and *SR*_max_=300 T/m/s, available frequency range for $N$=1, 2, and 3 were 30–60 Hz, 60–80 Hz, and 80–90Hz, respectively. Hence, for fixed-$T$ regime, we initially selected 30Hz-N1, 60Hz-N2, and 90Hz-N3 with the same gradient waveform duration before refocusing pulse. However, the optimized $t_{M}$ was different between gradient waveforms, leading to unmatched total waveform times (the sum of both durations before and after refocusing pulse + $t_{M}$), which are 77.3, 86.4, and 80.0 ms for 30Hz-N1, 60Hz-N2, and 90Hz-N3, respectively. For $N$ = 2, the gradient waveform at 65Hz yielded a $T$=79.9 ms, which is closer to the other two waveforms (30Hz-N1 and 90Hz-N3). Thus, we changed the frequency selection for the fixed-$T$ regime to 30Hz-N1, 65Hz-N2, and 90Hz-N3. For the fixed-$N$ regime, $N$=1 has the largest achievable frequency range (30 Hz) compared to $N$=2 (20 Hz) and $N$=3 (10 Hz); therefore, we selected $N$=1 for the fixed-$N$ regime. To ensure sufficient contrast between the fixed-$N$ gradient waveforms, the frequency step was set to 10 Hz, leading to the chosen waveforms of 30Hz-N1, 40Hz-N1, 50Hz-N1, and 60Hz-N1 for the fixed-$N$ regime.

In our study protocol design, we prioritized the gradient waveform to have the optimized $t_{M}$. The way how we acquired the optimized $t_{M}$ for each gradient waveform is described in **Supporting Information Methods 1**. Here we summarized some details. If we target “truly” fixed-$T$, we should have used the gradient waveforms of 30Hz-N1, 60Hz-N2, and 90Hz-N3 with the same $t_{M}$. To fix $T$ = 80 ms, we need to use the $t_{M}$=10.1 ms, which is the same value as the optimized $t_{M}$ for 90Hz-N3 (**Table 1**). The power spectrum of 60Hz-N2 with $t_{M}$=10.1 ms is very unbalanced (with a very asymmetric profile and a secondary side lobe of high amplitude), totally different from that of 90Hz-N3 (**Figure S7**). In contrast, with the optimized $t_{M}$ for each waveform, the power spectra are mostly balanced with similar profiles (two small side lobes next to the main lobe), while 30Hz-N1 could not achieve the fully balanced secondary lobes due to the use of non-optimized $t_{M}$=7.4 ms. The shortest achievable optimized $t_{M}$ for 30Hz-N1 is 41.7 ms, which leads to very long TE and low signal-to-noise ratio and is impractical to use. More details are illustrated in **Supporting Information Methods 1**.

Non-optimized $t_{M}$ (7.4 ms) used in 30Hz-N1 resulted in sudden drop between data points of 30Hz-N1 and 40Hz-N1 in **Figure 3B**. To plot a fitting curve for fixed-$N$ regime in **Figure 3B** (orange solid line), we calculated mean kurtosis (MK) using the gradient waveforms with optimal $t_{M}$ based on **Supporting Information Methods 1**. Empirically, the higher the frequency, the shorter the $t_{M}$. However, if the optimal $t_{M}$ is too short to accommodate the refocusing pulse (<7.4 ms) or too long (>40 ms) to be fitted within the TE (118 ms), we set the $t_{M}$ to 7.4 ms. Thus, for the gradient waveforms of $N$=1 with a peak frequency lower than 26.7 Hz, we use the same $t_{M}$ of 7.4 ms. For the fixed-$N$ regime, specifically, to explain the sudden drop of MK curve at the frequency of 26.7 Hz, we calculated the theoretical $\mathrm{MK}=\bar{K}_{var}\cdot h[q]$ of 30Hz-N1 waveform with different $t_{M}$ ranging from 7 to 42 ms. When we used the median values of model parameters within global WM and cortical GM, $\bar{K}_{var}\cdot h[q]$ monotonically decreased with increase of $t_{M}$ (**Figure S8**). This explains why the use of a short $t_{M}$ (7.4 ms) for the fixed-$N$=1 waveforms with peak frequencies <26.7 Hz leads to a higher MK value. As peak frequencies of 30Hz-N1 and 40Hz-N1 waveforms are 23.2 and 34 Hz, the sudden drop (at peak frequency 26.7 Hz) between data points of 30Hz-N1 and 40Hz-N1 was observed.

**Supporting Information Figure S1**


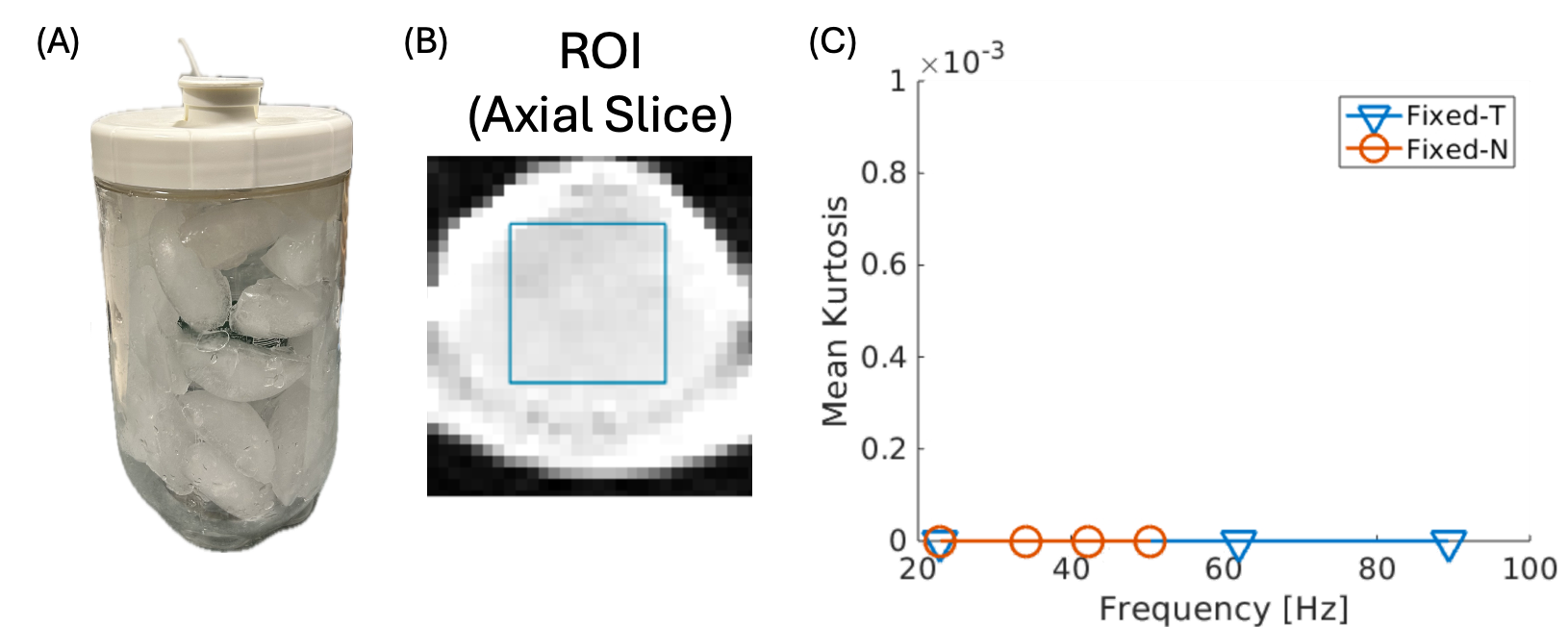


**Figure S1**. (A) A distilled water phantom (inner bottle) surrounded with ice (outer bottle). (B) A b=0 image in axial view with a region of interest of 30×30×30 mm^3^ cube (a blue rectangle centered at the inner water bottle). (C) For all six OGSE gradient waveforms (30Hz-N1, 40Hz-N1, 50Hz-N1, 60Hz-N1, 65Hz-N2, 90Hz-N3), median values of mean kurtosis (MK) across the ROI were almost zero (< 10^-5^) with no noticeable frequency-dependence.

**Supporting Information Figure S2**

**
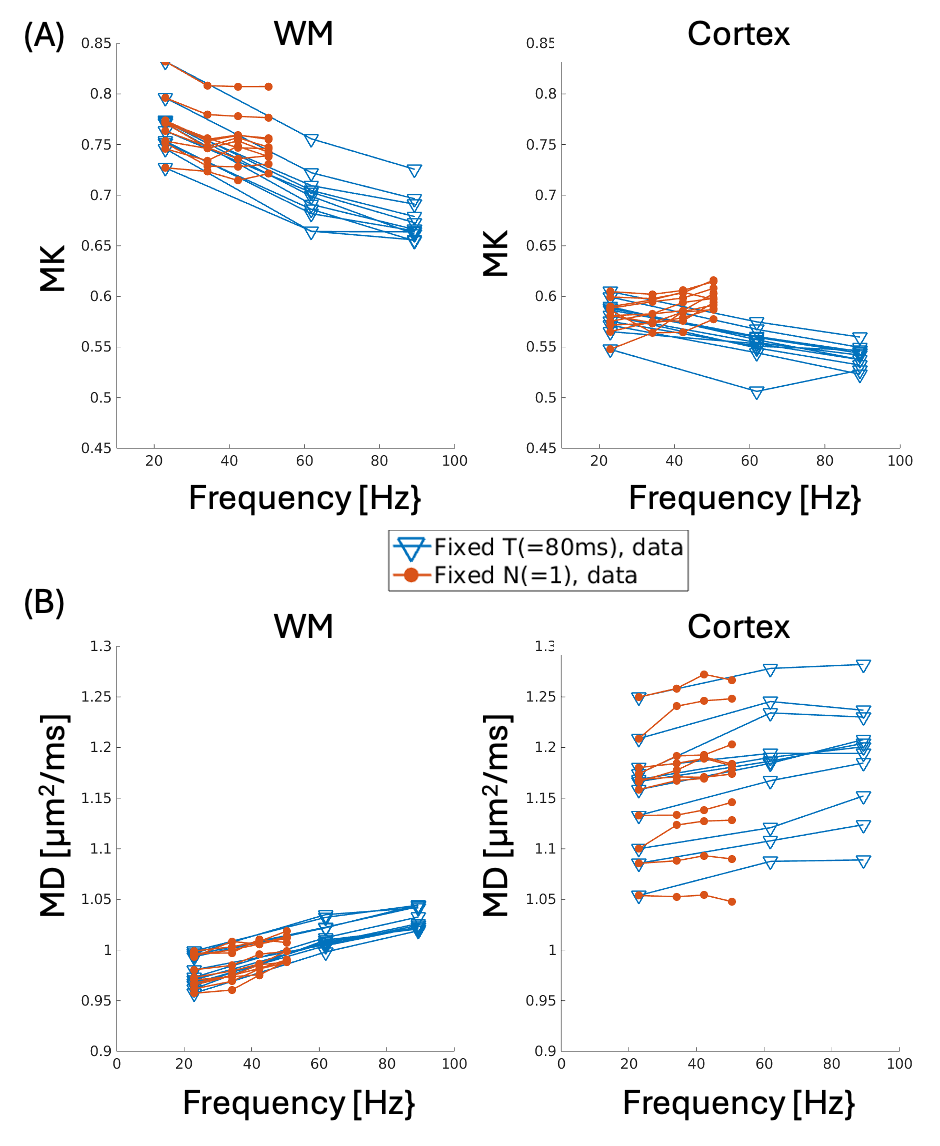
**

**Figure S2.** (A) Mean kurtoses and (B) mean diffusivities from all subjects in WM and global cortex for both fixed-T (blue triangles and solid lines) and fixed-N regime (orange dots and solid lines).

**Supporting Information Figure S3**

**
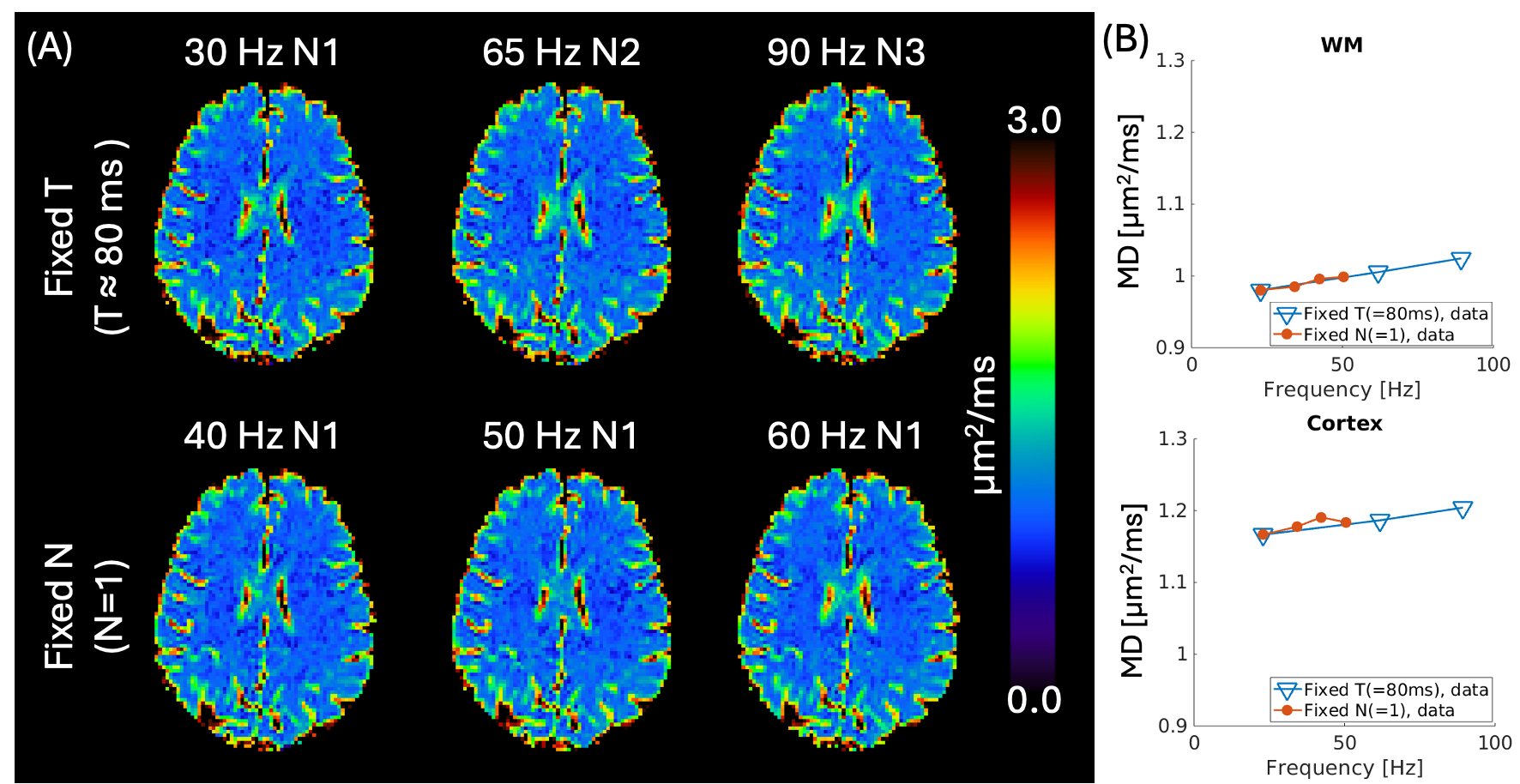
**

**Figure S3.** (A) Mean diffusivity (MD) maps in an axial view from a single subject in both fixed-T (first row) and fixed-N (second row) regimes. MD maps range from 0 to 3 μm^2^/ms. (B) ROI analysis of frequency-dependent MD in global WM and cortical GM of the same subject.

**Supporting Information Figure S4**

**Figure S4**. (A) A density scatter plot with y=x line (red solid line) and Pearson’s correlation coefficient (R) is displayed. (B) A Bland-Altman plot for all 7788 data points [11 subject, 6 gradient waveforms, and 118 regions of interest (ROIs)] with Lin’s concordance correlation coefficient (CCC) and root mean square error (RMSE) is displayed. (C) Bland-Altman plots for left and right ROIs are displayed with corresponding Lin’s CCC and RMSE. (D) Bland-Altman plots for gray and white matter (GM and WM) ROIs are displayed with corresponding Lin’s CCC and RMSE. A red solid line in the Bland-Altman plot represents a mean bias (<0.01), and black dashed lines represents upper and lower limit of agreement which are mean ± 1.96 × standard deviation (SD). Most data points are within the limit of agreement.

**Supporting Information Figure S5**

**
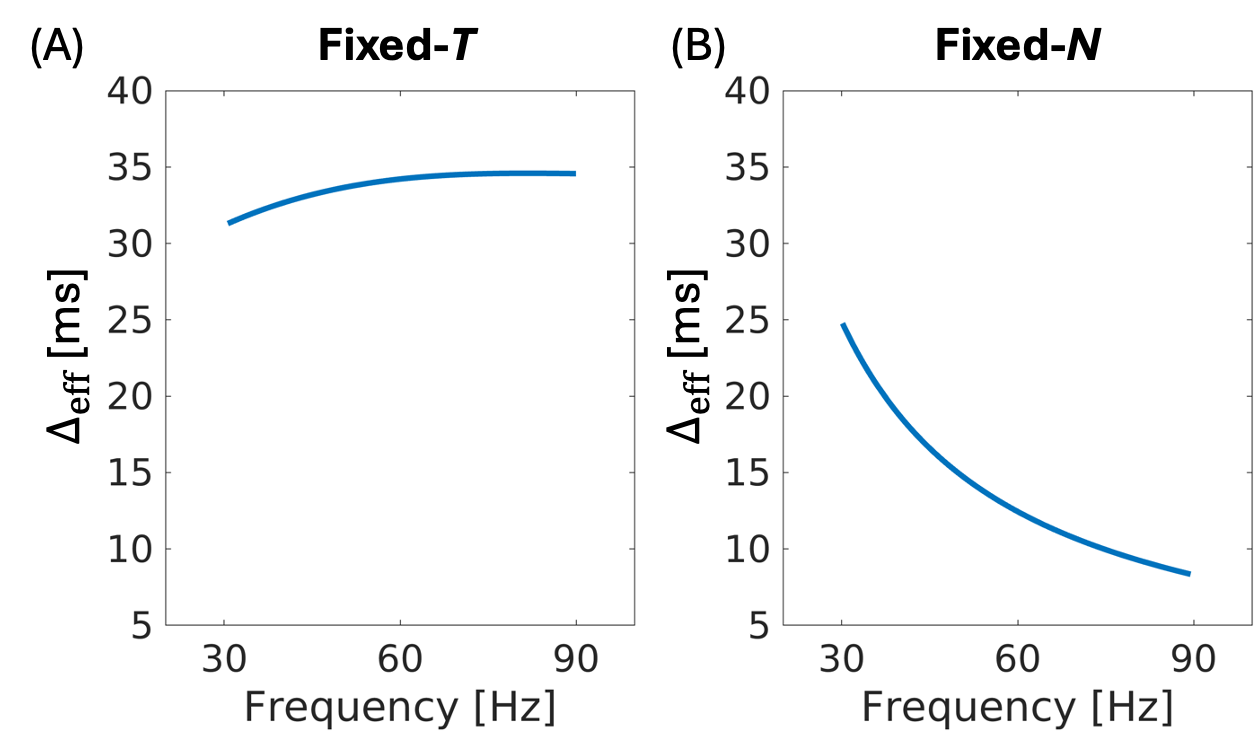
**

**Figure S5.** Effective diffusion times ($\Delta_{eff}$) were calculated in the frequency range of 30 – 90 Hz for both (A) fixed-$T$ and (B) fixed-$N$ regimes. $\Delta_{eff}$ slightly increases with increasing frequency in fixed-$T$ regime, whereas $\Delta_{eff}$ largely decreases with increasing frequency in fixed-$N$ regime.

**Supporting Information Figure S6**

**
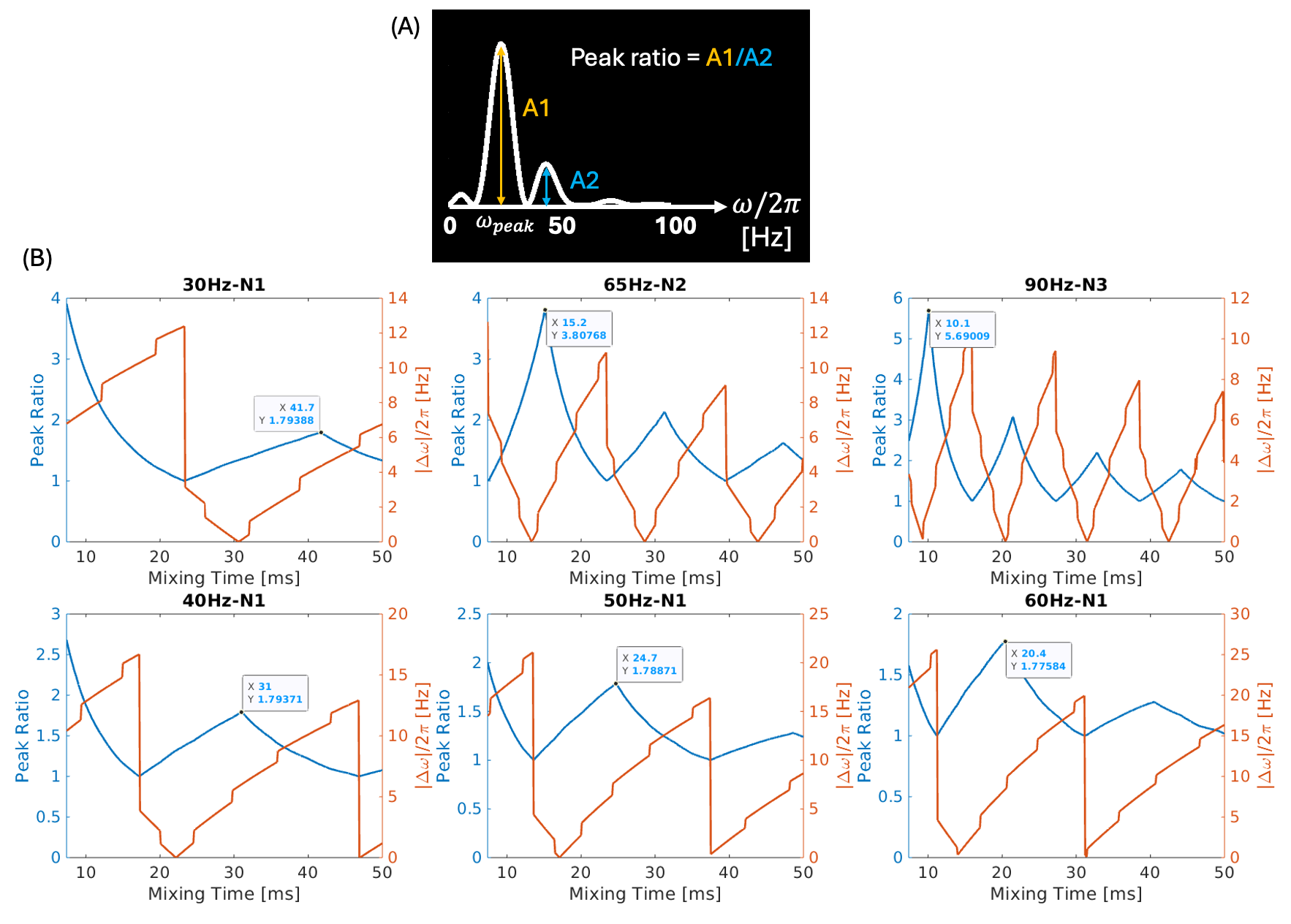
**

**Figure S6.** (A) A power spectrum showing a major lobe with an amplitude A1 and the secondary lobe with an amplitude of A2. (B) Peak ratios (= A1/A2) and frequency discrepancies between input and actual peak frequencies ($|\Delta\omega|/2\pi$) are depicted in blue and orange lines. For each gradient waveform, local maximum of peak ratio with the shortest applicable mixing time are also shown on each corresponding panel (e.g., mixing time = 10.1 ms for 90Hz-N3).

**Supporting Information Figure S7**

**
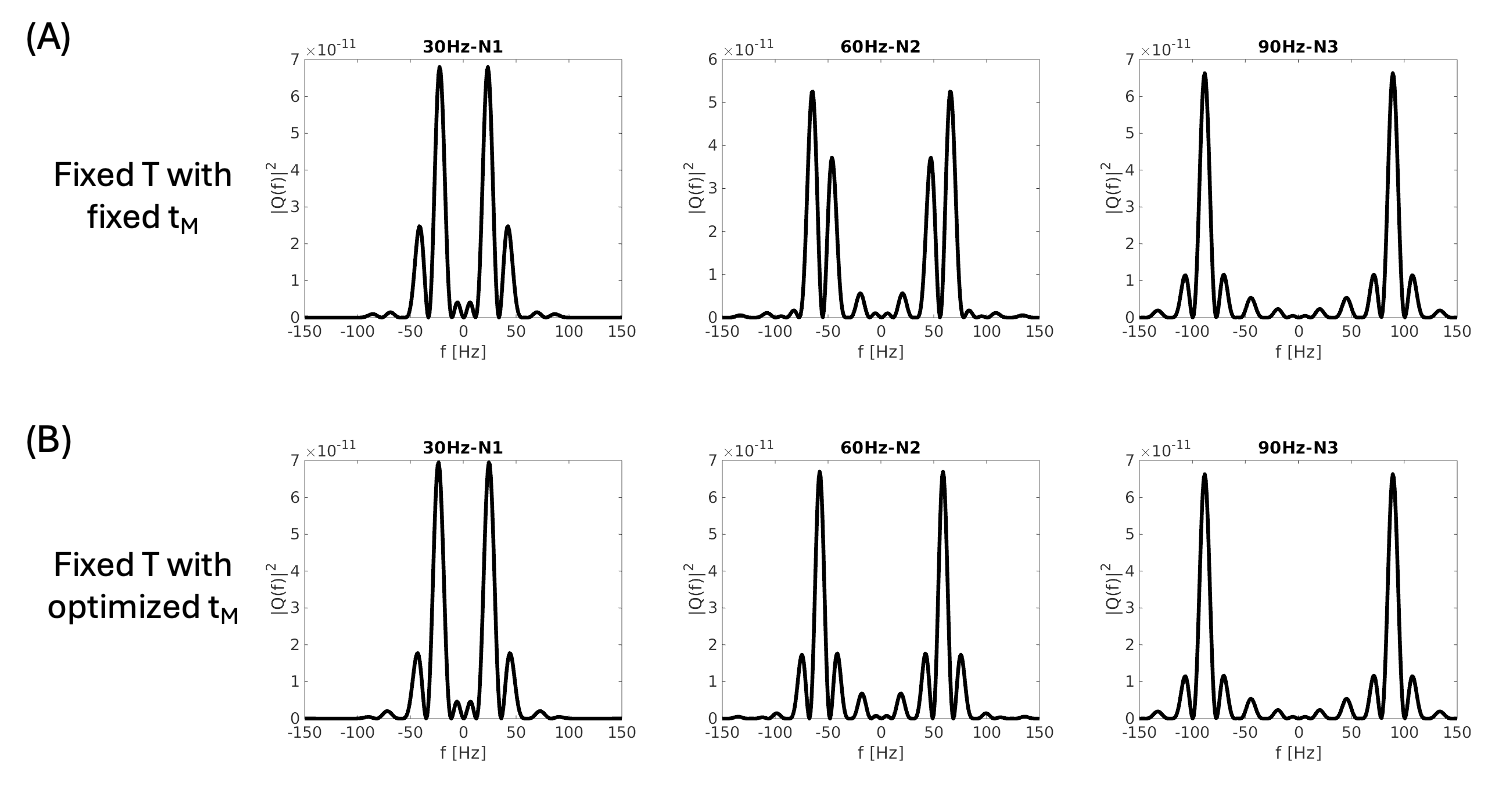
**

**Figure S7.** Power spectra of gradient waveforms of 30Hz-N1, 60Hz-N2, and 90Hz-N3 from left to right are plotted in the case of (A) a fixed total waveform time (*T*) of 80 ms with a fixed mixing time ($t_{M}$) of 10.1 ms and (B) approximately fixed *T* with optimized $t_{M}$.

**Supporting Information Figure S8**


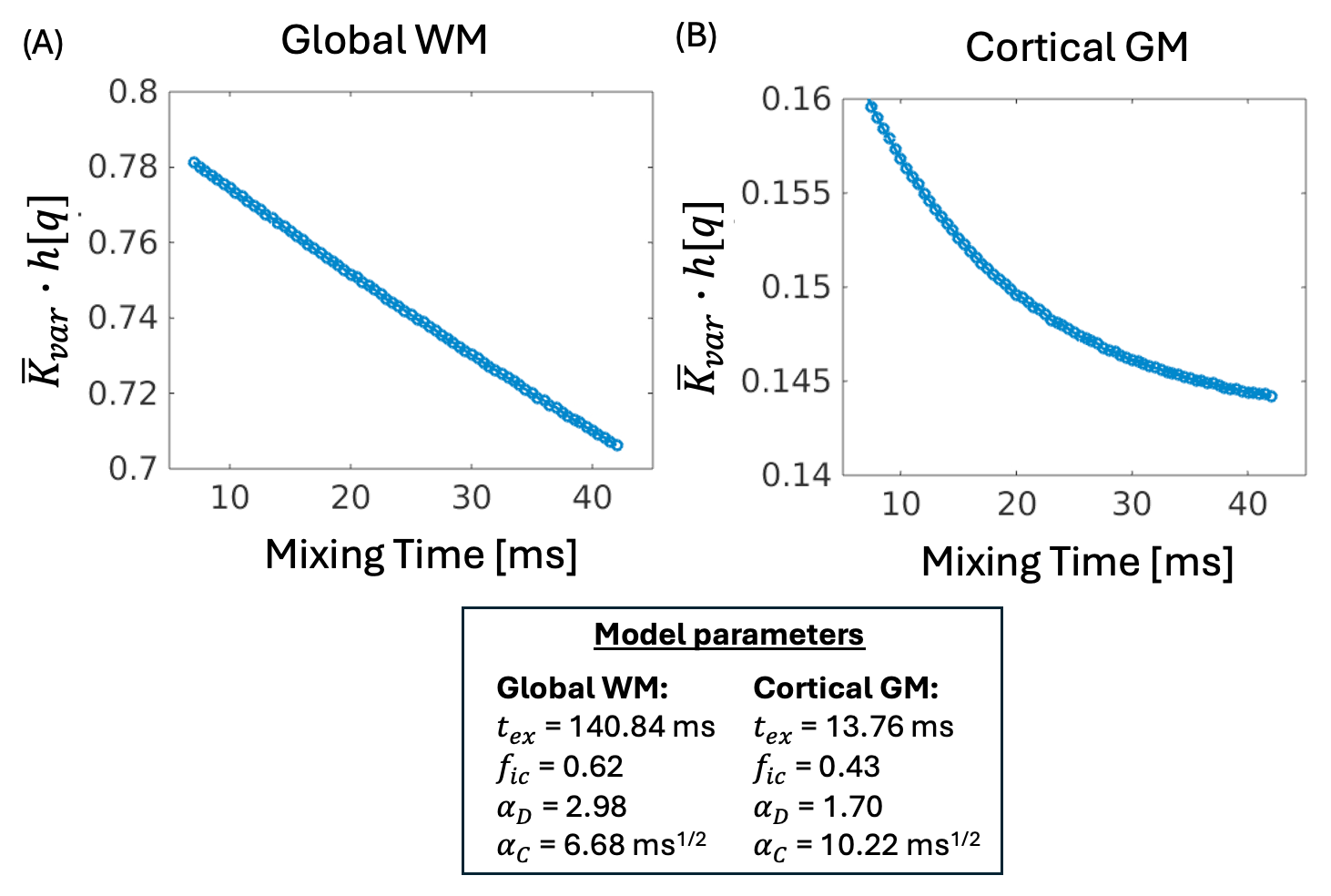


**Figure S8.** Relationships between overall mean kurtosis $\bar{K}_{var}\cdot h[q]$ and mixing time ($t_{M}$) are displayed based on typical values of model parameters in (A) white matter (WM) and (B) gray matter (GM). $\bar{K}_{var}\cdot h[q]$ monotonically decreased with increase of $t_{M}$ within the range of 7 – 42 ms. Model parameters for WM and GM are presented below.

**Supporting Information Table S1**

**Table S1.** **Regional Microstructural Parameters Estimated from the Adiabatic Kärger Model.** Median ± interquartile range of microstructure parameters ($t_{ex}$, $f_{ic}$, $\alpha_{D}$, $\alpha_{C}$, and $K_{\infty}$) in global white matter (WM) and four representative WM regions of interest (ROIs), as well as for global cortical GM and the frontal, parietal, temporal, and occipital lobes across all participants.

| **Fixed Regime**​ | $t_{ex}$ [ms] | ​$f_{ic}$ | $\alpha_{D}$ | $\alpha_{C}$ [ms^1/2^] | $K_{\infty}$ |
| --- | --- | --- | --- | --- | --- |
| WM | 140.61 ± 12.25 | ​0.62 ± 0.01 | 2.95 ± 0.30 | 6.67 ± 0.51 | 0.02 ± 0.01 |
| CC | 159.28 ± 16.53 | 0.61 ± 0.02 | 3.38 ± 1.15 | 6.23 ± 0.83 | 0.04 ± 0.02 |
| PLIC | 164.16 ± 16.53 | 0.64 ± 0.01 | 2.49 ± 0.36 | 7.39 ± 0.91 | 0.03 ± 0.03 |
| SCR | 163.52 ± 32.93​ | 0.65 ± 0.01 | 2.16 ± 0.55 | 7.74 ± 2.24 | 0.01 ± 0.01 |
| SLF | 162.41 ± 10.09 ​ | 0.65 ± 0.01 | 2.35 ± 0.26 | 7.15 ± 1.18 | 0.01 ± 0.03 |
| Cortex | 13.32 ± 6.54 | 0.42 ± 0.02 | 1.71 ± 0.63 | 10.22 ± 1.69 | 0.43 ± 0.03 |
| Frontal lobe | 12.53 ± 7.62 | 0.42 ± 0.04 | 1.81 ± 0.61 | 9.91 ± 1.43 | 0.42 ± 0.03 |
| Parietal lobe | 14.45 ± 5.42 | 0.42 ± 0.04 | 1.81 ± 0.29 | 10.11 ± 1.08 | 0.43 ± 0.02 |
| Temporal lobe | 12.29 ± 7.32 | 0.43 ± 0.05 | 1.76 ± 0.57 | 9.84± 1.11 | 0.42 ± 0.06 |
| Occipital lobe | 20.05 ± 3.19 | 0.44 ± 0.08 | 1.52 ± 0.37 | 10.24 ± 1.07 | 0.38 ± 0.06 |
| Abbreviations: $t_{ex}$, water exchange time; $f_{ic}$, intra-cellular signal fraction; $\alpha_{D}$, extra-cellular tortuosity; $\alpha_{C}$, frequency-dependent factor; $K_{\infty}$, asymptotic kurtosis value in the long-time limit; WM, white matter; CC, corpus callosum; PLIC, posterior limb of internal capsule; SCR, superior corona radiata; SLF, superior longitudinal fasciculus. | | | | | |

**Supporting Information Table S2**

**Table S2.** **All combination of four gradient waveforms with corresponding accumulated scores and median values of coefficient of variance (*cv*) across all regions of interest (ROI)**. Accumulated scores and median $cv$ are displayed for four model parameters, including $t_{ex}$, $f_{ic}$, $\alpha_{D}$, and $\alpha_{C}$.

| **Combination of  gradient waveforms** | **​Accumulated Scores** | | | | **Median *cv* across all ROIs** | | | |
| --- | --- | --- | --- | --- | --- | --- | --- | --- |
|  | $t_{ex}$ [ms] | $f_{ic}$ | $\alpha_{D}$ | $\alpha_{C}$ [ms^1/2^] | $t_{ex}$ [ms] | $f_{ic}$ | $\alpha_{D}$ | $\alpha_{C}$ [ms^1/2^] |
| 30Hz-N1, 40Hz-N1, 50Hz-N1, 60Hz-N1 | 132 | 143 | 248 | 170 | 0.63 | 0.36 | 1.29 | 1.14 |
| 30Hz-N1, 40Hz-N1, 50Hz-N1, 65Hz-N2 | 154 | 134 | 145 | 161 | 0.57 | 0.35 | 1.30 | 1.23 |
| 30Hz-N1, 40Hz-N1, 60Hz-N1, 65Hz-N2 | 160 | 141 | 160 | 134 | 0.52 | 0.36 | 1.48 | 1.26 |
| 30Hz-N1, 50Hz-N1, 60Hz-N1, 65Hz-N2 | 182 | 146 | 195 | 166 | 0.71 | 0.34 | 1.26 | 1.13 |
| 40Hz-N1, 50Hz-N1, 60Hz-N1, 65Hz-N2 | 118 | 118 | 176 | 113 | 0.60 | 0.43 | 1.50 | 1.58 |
| 30Hz-N1, 40Hz-N1, 50Hz-N1, 90Hz-N3 | 135 | 163 | 110 | 137 | 0.62 | 0.33 | 1.55 | 1.29 |
| 30Hz-N1, 40Hz-N1, 60Hz-N1, 90Hz-N3 | 172 | 170 | 151 | 188 | 0.65 | 0.34 | 1.90 | 1.23 |
| 30Hz-N1, 50Hz-N1, 60Hz-N1, 90Hz-N3 | 153 | 168 | 134 | 164 | 0.66 | 0.29 | 1.59 | 1.03 |
| 40Hz-N1, 50Hz-N1, 60Hz-N1, 90Hz-N3 | 168 | 141 | 105 | 128 | 0.63 | 0.36 | 1.42 | 1.19 |
| 30Hz-N1, 40Hz-N1, 65Hz-N2, 90Hz-N3 | 132 | 150 | 117 | 145 | 0.67 | 0.39 | 1.79 | 1.38 |
| 30Hz-N1, 50Hz-N1, 65Hz-N2, 90Hz-N3 | 144 | 142 | 125 | 151 | 0.58 | 0.33 | 1.55 | 1.11 |
| 40Hz-N1, 50Hz-N1, 65Hz-N2, 90Hz-N3 | 163 | 145 | 113 | 158 | 0.61 | 0.34 | 1.77 | 1.19 |
| 30Hz-N1, 60Hz-N1, 65Hz-N2, 90Hz-N3 | 153 | 136 | 114 | 153 | 0.63 | 0.34 | 2.20 | 1.20 |
| 40Hz-N1, 60Hz-N1, 65Hz-N2, 90Hz-N3 | 145 | 128 | 65 | 114 | 0.66 | 0.39 | 2.01 | 1.39 |
| 50Hz-N1, 60Hz-N1, 65Hz-N2, 90Hz-N3 | 108 | 149 | 93 | 113 | 0.64 | 0.34 | 2.14 | 1.19 |
| Abbreviations: $t_{ex}$, water exchange time; $f_{ic}$, intra-cellular signal fraction; $\alpha_{D}$, extra-cellular tortuosity; $\alpha_{C}$, frequency-dependent factor | | | | | | | | |

**References**

1. Does MD, Parsons EC, Gore JC. Oscillating gradient measurements of water diffusion in normal and globally ischemic rat brain. *Magn Reson Med*. 2003;49(2):206-215.

2. Novikov DS, Jensen JH, Helpern JA, Fieremans E. Revealing mesoscopic structural universality with diffusion. *Proc Natl Acad Sci*. 2014;111(14):5088-5093. doi:<https://doi.org/10.1073/pnas.1316944111>

3. Fieremans E, Burcaw LM, Lee H-H, Lemberskiy G, Veraart J, Novikov DS. In vivo observation and biophysical interpretation of time-dependent diffusion in human white matter. *Neuroimage*. 2016;129:414-427. doi:<https://doi.org/10.1016/j.neuroimage.2016.01.018>

4. Lee H-H, Papaioannou A, Kim S-L, Novikov DS, Fieremans E. A time-dependent diffusion MRI signature of axon caliber variations and beading. *Commun Biol*. 2020;3(1):354. doi:<https://doi.org/10.1038/s42003-020-1050-x>

5. Lee H-H, Papaioannou A, Novikov DS, Fieremans E. In vivo observation and biophysical interpretation of time-dependent diffusion in human cortical gray matter. *Neuroimage*. 2020;222:117054. doi:<https://doi.org/10.1016/j.neuroimage.2020.117054>

6. Neuman C. Spin echo of spins diffusing in a bounded medium. *J Chem Phys*. 1974;60(11):4508-4511.

7. Novikov DS, Kiselev VG. Surface-to-volume ratio with oscillating gradients. *J Magn Reson*. 2011;210(1):141-145.

8. Van AT, Holdsworth SJ, Bammer R. In vivo investigation of restricted diffusion in the human brain with optimized oscillating diffusion gradient encoding. *Magn Reson Med*. 2014;71(1):83-94.
